# Expression and chromatin structures of cellulolytic enzyme gene regulated by heterochromatin protein 1

**DOI:** 10.1186/s13068-016-0624-9

**Published:** 2016-10-03

**Authors:** Xiujun Zhang, Yinbo Qu, Yuqi Qin

**Affiliations:** 1 National Glycoengineering Research Center and State Key Lab of Microbial Technology, Shandong University, Jinan, 250100 China; 2 Shandong Provincial Key Laboratory of Carbohydrate Chemistry and Glycobiology, Shandong University, Jinan, 250100 China

**Keywords:** HP1, *Penicillium oxalicum*, CHART-PCR, Cellulolytic enzyme, Chromatin

## Abstract

**Background:**

Heterochromatin protein 1 (HP1, homologue HepA in *Penicillium oxalicum*) binding is associated with a highly compact chromatin state accompanied by gene silencing or repression. HP1 loss leads to the derepression of gene expression. We investigated HepA roles in regulating cellulolytic enzyme gene expression, as an increasingly number of studies have suggested that cellulolytic enzyme gene expression is not only regulated by transcription factors, but is also affected by the chromatin status.

**Results:**

Among the genes that exhibited significant differences between the *hepA* deletion strain (Δ*hepA*) and the wild type (WT), most (95.0 %) were upregulated in Δ*hepA* compared with WT. The expression of the key transcription factor for cellulolytic enzyme gene (e.g., repressor CreA and activator ClrB) increased significantly. However, the deletion of *hepA* led to downregulation of prominent extracellular cellulolytic enzyme genes. Among the top 10 extracellular glycoside hydrolases (Amy15A, Amy13A, Cel7A/CBHI, Cel61A, Chi18A, Cel3A/BGLI, Xyn10A, Cel7B/EGI, Cel5B/EGII, and Cel6A/CBHII), in which secretion amount is from the highest to the tenth in *P*. *oxalicum* secretome, eight genes, including two amylase genes (*amy15A* and *amy13A*), all five cellulase genes (*cel7A*/*cbh1*, *cel6A*/*cbh2*, *cel7B*/*eg1*, *cel5B*/*eg2*, and *cel3A*/*bgl1*), and the cellulose-active LPMO gene (*cel61A*) expression were downregulated. Results of chromatin accessibility real-time PCR (CHART-PCR) showed that the chromatin of all three tested upstream regions opened specifically because of the deletion of *hepA* in the case of two prominent cellulase genes *cel7A/cbh1* and *cel7B/eg1*. However, the open chromatin status did not occur along with the activation of cellulolytic enzyme gene expression. The overexpression of *hepA* upregulated the cellulolytic enzyme gene expression without chromatin modification. The overexpression of *hepA* remarkably activated the cellulolytic enzyme synthesis, not only in WT (~150 % filter paper activity (FPA) increase), but also in the industry strain RE-10 (~20–30 % FPA increase).

**Conclusions:**

HepA is required for chromatin condensation of prominent cellulase genes. However, the opening of chromatin mediated by the deletion of *hepA* was not positively correlated with cellulolytic enzyme gene activation. HepA is actually a positive regulator for cellulolytic enzyme gene expression and could be a promising target for genetic modification to improve cellulolytic enzyme synthesis.

**Electronic supplementary material:**

The online version of this article (doi:10.1186/s13068-016-0624-9) contains supplementary material, which is available to authorized users.

## Background

Lignocellulolytic enzymes are industrially important enzymes, particularly in the textile or paper industry. In the production of cellulosic ethanol, lignocellulolytic enzymes are applied to break down lignocellulosic material to release d-glucose, which can subsequently be used in the sugar-to-ethanol fermentation by yeast. The costs of the necessary enzymes during this process have a huge influence on the price and competitiveness of the end-product [1]. Some filamentous fungi, such as *Trichoderma reesei* [2], *Aspergillus niger* [3], *Neurospora crassa* [4], and *Penicillium oxalicum* [5], can secrete a complex arsenal of enzymes that synergistically deconstructs lignocellulosic material. Research on the regulation of cellulolytic enzyme gene expression may be very useful in increasing the production of these enzymes in their native hosts.

Cellulolytic enzyme production is tightly controlled at the transcriptional level in fungi. Several transcriptional activators (i.e., orthologous XlnR/Xyr1 and ClrB/Clr2) [6, 7] and repressors (i.e., orthologous CreA/Cre1) [8] participate in the process. Transcription factors are currently thought to induce the reorganization of local chromatin in eukaryotes; activators recruit nucleosome modifiers that help transcriptional machinery bind at the promoter or initiate transcription [9]. Two types of nucleosome modifiers exist, those that remodel the nucleosomes, such as ATP-dependent activity of SWI/SNF [10], and those that add chemical groups to the tails of histone, such as histone methyltransferases or histone acetyltransferases [11]. This modification can “loosen” the chromatin structure, rendering it accessible and capable of binding the transcriptional machinery [12]. For example, the helicase gene *snf2*, which is a component of yeast Swi/Snf multisubunit chromatin remodeling complex, was found to be upregulated significantly in *T. reesei* Δ*cre1* mutant and thought to be involved in the cellulase gene repression by Cre1 at a high growth rate [13]. Another chromatin-associated protein (*T. reesei* ID 107641), which has a bromodomain, a module found in many chromatin-associated proteins and which plays a key role in chromatin remodeling, was reported to bear the highest expression in *T. reesei* Δ*cre1* when the strain was cultivated in cellulose and is thought to result in an alteration of chromatin structure that consequently change the expression of genes related to lignocelluloses degradation [14]. Histone acetyltransferase Gcn5 has also been proven to be required in the induction expression of cellulase genes in *T. reesei*, with acetylation of K9 and K14 of histone H3 in the cellulase gene promoter dramatically affected in the absence of Gcn5 [15]. The lysines of histones H3 are usually acetylated in transcriptionally active chromatin, and usually hypoacetylated in transcriptionally repressed or silent chromatin [16]. The lack of acetylation enables the mono-, di-, or tri-methylation of H3K9 (H3K9me) by histone methyltransferase, such as Clr4 [17]. This histone modification could be recognized by the heterochromatin protein 1 (HP1) [18].

HP1, a small non-histone chromosomal protein, was first identified in *Drosophila melanogaster* as a dominant suppressor of position-effect variegation (PEV) on heterochromatin gene silencing [19]. HP1 protein binding is always associated with highly compact chromatin state accompanied by gene silencing or repression, whereas its loss leads to the derepression of gene expression. The best-studied fungal HP1 homologues in fungi were the *Schizosaccharomyces pombe* SWI6 (reviewed in [20]), *N*. *crassa* Hpo [21], and *Aspergillus nidulans* HepA [22]. *N*. *crassa* Hpo specifically binds H3K9me3, recruits DNA methyltransferase DIM-2, and forms distinct DNA methylation complexes involved in gene repression or gene silencing [21]. *S*. *pombe* SWI6 interacts with and recruits H3K9 methyltransferase Clr4, and plays a role in the establishment and maintenance of heterochromatin [18]. The heterochromatinization mediated by HepA/Clr4 was counteracted by LaeA (ortholog of Lae1) in *A. nidulans* [22]. LaeA is a putative methyltransferase considered to be a positive regulator for glycoside hydrolase gene expression because its overexpression improves cellulase gene transcription, whereas a complete loss of expression of all seven cellulases and auxiliary factors for cellulose degradation was observed in the *T. reesei lae1* deletion strain [23]. The deletion of *hepA* upregulated *laeA* expression; whereas LaeA prevented HepA binding and the formation of repressive chromatin [22], suggesting that HepA might be involved in cellulolytic enzyme gene expression. Thiago also found that HP1 **gene** expression was downregulated significantly when *T. reesei* QM6a was cultivated in a sophorose medium, which is an induction medium for cellulolytic enzyme gene expression, compared with that in glucose repression medium [24]. Therefore, a possible speculation could be that HepA might be a negative regulator for cellulolytic enzyme gene expression.

To determine whether HepA participates in gene expression, especially in cellulolytic enzyme gene expression in filamentous fungi, the *hepA* gene was deleted and overexpressed in *P. oxalicum*, which is a strain used in industrial-scale glycoside hydrolase production [25, 26]. As no report has been made on the positive roles of HP1 in filamentous fungi, it is rather surprising that the deletion of *hepA* indicated the repression of cellulolytic enzyme gene, whereas the overexpression of *hepA* exhibited obvious activation of cellulolytic enzyme synthesis not only in a wild-type (WT) strain, but also in an industry strain. The unexpected results showed that HepA is actually a positive regulator for cellulolytic enzyme gene expression. We found that HepA is required for chromatin condensation using chromatin accessibility real-time PCR (CHART-PCR). However, the opening of chromatin in the absence of HepA did not occur with the activation of gene expression, suggesting that chromatin condensation or decondensation in the core promoter region alone does not repress or activate gene expression.

## Results

### Identification of *P. oxalicum* HepA

**Fig. 1:**
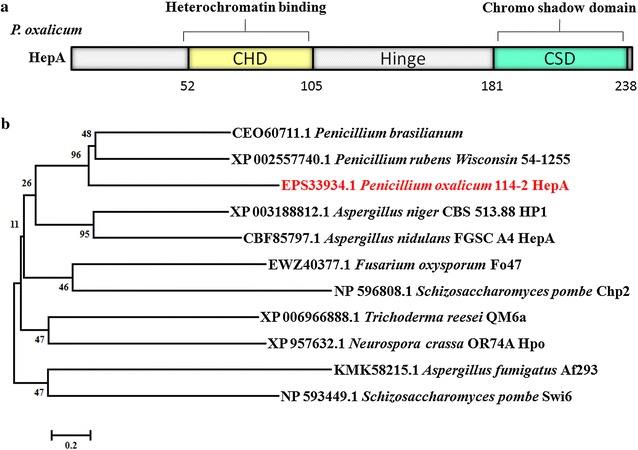
Schematic reprsentation and phylogenetic analysis of *P. oxalicum* HepA protein. **a** Schematic representation of HepA. HepA protein consists of chromodomain (CHD) at the N-terminus and chromoshadow domain (CSD) at the C-terminus separated by the hinge region. The different functional roles of the different domains are indicated. **b** Phylogenetic analysis of HepA/HP1. The tree was constructed using neighbor joining in MEGA 5.0 with 500 bootstrap replicates (coefficients are indicated below the respective nodes). Gaps in the alignment were not considered

Similar to *S*. *pombe* and *N*. *crassa*, *P*. *oxalicum* contains only one HP1 homologue, HepA. *P*. *oxalicum* HepA contains 241 amino acids of two conserved domains according to the Pfam analysis [27]. One is a 54-amino-acid N-terminal chromatin organization modifier domain (CHD), and the other is a 58-amino-acid C-terminal chromo shadow domain (CSD). These domains are joined by a flexible hinge (Fig. 1a). The CHD is involved in the interaction with H3K9-methylated nucleosomes required for chromatin binding, whereas CSD mainly mediates interaction with other heterochromatin proteins [28]. HP1 is phylogenetically conserved and found in almost all eukaryotes, with the exception of budding yeast. *P*. *oxalicum* HepA exhibits a limited overall identity (28 %) to *S*. *pombe* SWI6, an identity (35 %) to *N*. *crassa* Hpo, and a higher identity (43 % identity) to *A. nidulans* HepA. The phylogenetic trees indicate the evolution of HepA (Fig. 1b).

### Diminished cellulolytic halo was observed around the Δ*hepA* colony

**Fig. 2:**
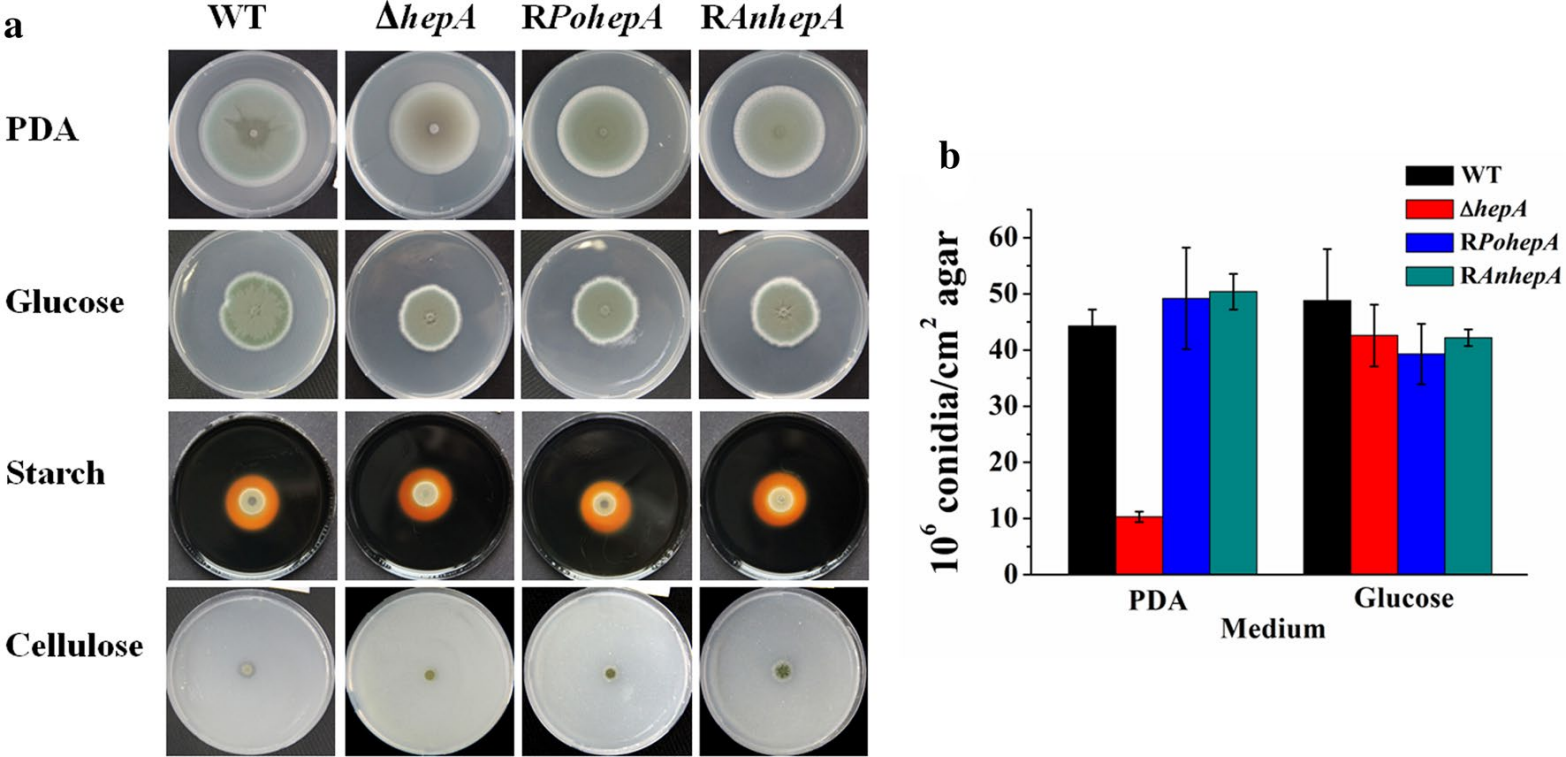
Colony morphology and conidiation of WT, Δ*hepA*, and recomplement strains. **a** Colony morphology of 5-day-old cultures for WT, Δ*hepA*, and the recomplement strains on PDA or VMM with 2 % glucose, 2 % starch, and 2 % cellulose at 30 °C. The 1 μL conidia solutions of WT, Δ*hepA*, R*pohepA*, and R*AnhepA* were dropped onto the agar at a density of 10^6^ conidia mL^−1^. **b** Levels of conidiation on PDA or Vogel’s salt agar with 2 % glucose. Plates were incubated at 30 °C for 4 days, and 5-mm diameter colony agar plugs in triplicate were sampled for each strain. The number of conidia was determined using a hemocytometer

The Δ*hepA* colony appeared greenish-brown compared with the dark-green WT and complemented strains when the strains were grown on potato dextrose agar (PDA) (Fig. 2a). The levels of conidiation in the Δ*hepA* mutant were significantly reduced (~23.3 % of WT) after five days of cultivation on PDA (Fig. 2b). Either expressions of *P. oxalicum hepA* (R*PohepA*) or *A. nidulans hepA* (R*AnhepA*) could recomplement the defect in Δ*hepA* (Fig. 2), indicating that the biological roles of *A. nidulans* HepA and *P. oxalicum* HaeA were well-conserved. The radial growth and conidiation of Δ*hepA* were identical to that of the WT when the strains were grown on VMM + glucose agar, which is in contrast with the *S*. *pombe* and *N*. *crassa* studied so far, in which HP1 deletion was shown to affect viability strongly. The mutations in *N*. *crassa* Hpo showed pronounced defects in asexual spore and aerial hyphae formation [29]. No cellulolytic halo was observed around the Δ*hepA* colony when the strains were grown on VMM + cellulose agar, whereas a clear cellulolytic halo was found around the WT colony, suggesting that the deletion of *hepA* suppressed cellulolytic enzyme formation.

### Transcriptome data showed that deletion of *hepA* led to downregulation of prominent extracellular cellulolytic enzyme genes

The unexpected observation of diminished cellulolytic halo around the Δ*hepA* colony led us to exploit the role of HepA in regulating cellulolytic enzyme gene expression. WT and Δ*hepA* were cultivated in cellulose and wheat bran medium, which is an optimum medium for cellulolytic enzyme formation, to obtain a global view of the effect of *hepA* deletion on gene expression, especially the cellulolytic enzyme gene expression. The mRNAs from WT and Δ*hepA* at cultivation time of 48 h were subjected to high-throughput Illumina sequencing (Realbio, Shanghai, China) to obtain the transcriptome of WT and Δ*hepA*. More than 10 million reads were obtained for each sample. The copy number of unambiguous reads (tags mapped to one single gene) for each gene was normalized to reads per kilobases per million reads (RPKM) clean reads. Saturation analysis indicated that the capacity of the two libraries approached saturation (Additional file 1: Fig. S1). A genome-wide transcriptome analysis revealed the extensive expression of the entire *P. oxalicum* genome. Among the 10,021 protein-coding genes predicted in the genome database, 9016 (90.0 %) and 9361 (93.4 %) genes were expressed in WT and Δ*hepA*, respectively. The genes of significantly differential expression levels were identified through a significance test with combined thresholds (FDR ≤ 0.001 and fold change ≥2) [30]. Blast2GO was used for the function enrichment analysis of gene sets with a threshold of FDR ≤ 0.05 [31].

**Fig. 3:**
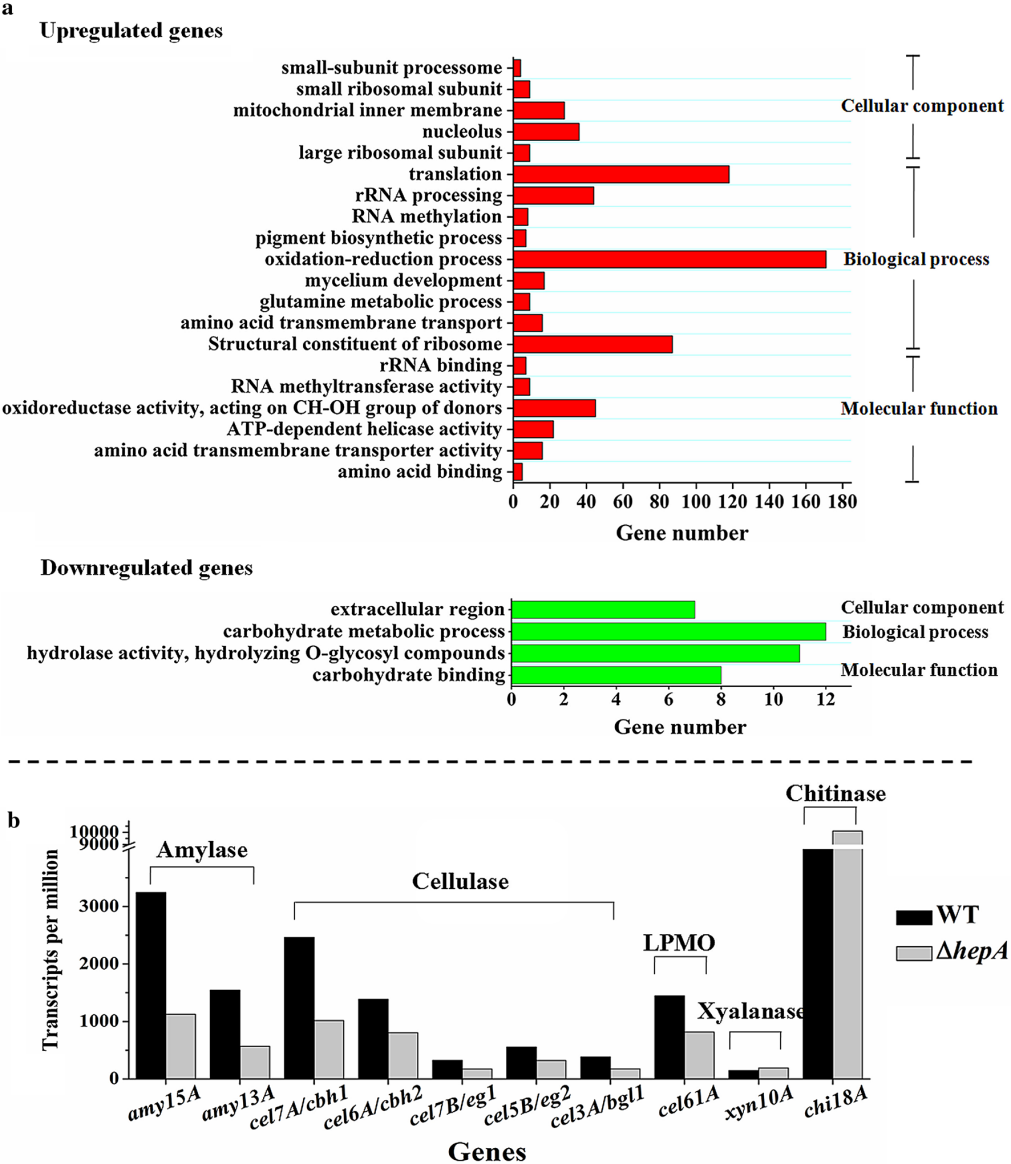
Genome-wide transcriptome analysis for WT and Δ*hepA*. WT and Δ*hepA* were cultivated in cellulose and wheat bran medium, which are optimum media for cellulolytic enzyme formation. mRNA from WT and Δ*hepA* at cultivation time of 48 h was collected and subjected to high-throughput Illumina sequencing. **a** Function enrichment analysis of upregulated or downregulated (≥twofold, FDR < 0.001) gene sets in Δ*hepA* compared with WT by Blast2GO with the threshold at FDR ≤ 0.05. *Red bars* function enrichment analysis of upregulated gene sets; *green bars* function enrichment analysis of downregulated gene sets. **b** Expression levels of the top 10 extracellular glycoside hydrolase genes. The copy number of unambiguous transcripts for each gene was normalized to RPKM (reads per kilobases per million reads)

Transcriptome data showed that the expression levels of 1654 genes exhibited significant differences (twofold or greater, FDR < 0.001) between the Δ*hepA* and WT. In the regulon, 1571 genes (95.0 %) were upregulated in Δ*hepA* compared with the WT. As expected, the vast majority of genes expression was upregulated in Δ*hepA* because HepA othologs (HP1 and Swi6) are always associated with gene repression or silencing. HP1 targeting results in a quantifiable degree of chromatin condensation and repressed genes, whereas the loss of HP1 disrupts repression completely [32]. GO enrichment analysis revealed that the upregulated genes in Δ*hepA* were involved mainly in amino acid binding, amino acid transmembrane transporter activity, ATP-dependent helicase activity, oxidoreductase activity acting on CH–OH group of donors, RNA methyltransferase activity, rRNA binding, and structural constituent of ribosome compared with that in WT (Fig. 3a, red bars). The GO terms which included most abundant genes in cellular component, biological process, and molecular function are translation, oxidation–reduction process, and oxidoreductase activity, respectively. For example, 118 ribosomal protein genes and 44 rRNA processing genes were enriched (Fig. 3; Additional file 2: Table S1), suggesting possible upregulation of protein synthesis in Δ*hepA*. The upregulated genes (GO category: molecular function) and their predicted functions are listed in the Additional file 2: Table S1.

In contrast to most genes in the regulon that showed upregulation, 83 genes (5.0 %) were downregulated in Δ*hepA*. Interestingly, GO enrichment analysis revealed that downregulated genes in Δ*hepA* were involved mainly in carbohydrate binding and hydrolase activity (Fig. 3a, green bars). The downregulated genes (GO category: molecular function) and their predicted functions are listed in Table 1. When *P. oxalicum* was cultivated on cellulose and wheat bran-containing media, the secretome data revealed that the top 10 extracellular glycoside hydrolases were glucoamylase Amy15A (PDE_09417, Genbank No. EPS34453.1), α-amylase Amy13A (PDE_01201, Genbank No. EPS26265.1), cellobiohydrolyase Cel7A/CBHI (PDE_07945, Genbank No. EPS32984.1), lytic polysaccharide monooxygenases (LPMO) Cel61A (PDE_05633, Genbank No. EPS30681.1), chitinase Chi18A (PDE_08122, Genbank No. EPS33160.1), β-glucosidase Cel3A/BGLI (PDE_02736, Genbank No. EPS27792.1), xylanase Xyn10A (PDE_08094, Genbank No. EPS33132.1), endoglucanase Cel7B/EGI (PDE_07929, Genbank No. EPS32968.1), endoglucanase Cel5B/EGII (PDE_09226, Genbank No. EPS34262.1), and cellobiohydrolyase Cel6A/CBHII (PDE_07124, Genbank No. EPS32164.1). Their products accounted for 28.9, 11.0, 9.6, 5.6, 4.9, 3.4, 3.3, 2.5, 1.9, and 1.5 % of the total extracellular protein of *P. oxalicum*, respectively [33]. Among the top 10 proteins (Amy15A, Amy13A, Cel7A/CBHI, Cel61A, Chi18A, Cel3A/BGLI, Xyn10A, Cel7B/EGI, Cel5B/EGII, and Cel6A/CBHII) in which secretion amount is from the highest to the tenth in *P*. *oxalicum* secretome, the top 3 extracellular glycoside hydrolases encoding genes (*amy15A*, *amy13A*, and *cel7A*/*cbh1*) and another β-glucosidase encoding gene (*cel3A*/*bgl1*), were found in Table 1 (the yellow background). Indeed, the transcriptome data showed that among the top 10 extracellular glycoside hydrolases, eight genes included two amylase genes (*amy15A* and *amy13A*), all five cellulase genes (*cel7A*/*cbh1*, *cel6A*/*cbh2*, *cel7B*/*eg1*, *cel5B*/*eg2*, and *cel3A*/*bgl1*), and a cellulose-active LPMO gene (*cel61A*) expression were downregulated (Fig. 3b) in Δ*hepA*, suggesting that the deletion of *hepA* led to the downregulation of prominent extracellular cellulolytic enzyme genes. A total of 28 non-redundant secondary metabolic gene clusters, including 36 backbone enzymes, were predicted by SMURF in *P. oxalicum* [5]. In addition to downregulated extracellular cellulolytic enzyme genes, gene expression in one of secondary metabolic gene clusters (cluster 15, from PDE_04008 to PDE_04029) was observed downregulated remarkably (Additional file 3: Fig. S2), while the other clusters were not regulated in Δ*hepA*. However, the function or biosynthesis product of clusters 15 was not determined yet.

**Table 1:** List of downregulated genes (≥twofold, FDR < 0.05) in Δ*hepA* compared with WT with significantly enriched GO terms (GO category: molecular function)

GO-ID	Term	Gene ID (locus_tag)	Description of putative *P. oxalicum* ORF
GO:0030246	Carbohydrate binding	*PDE_01201*	*Alpha-amylase Amy13A*
		PDE_01302	Endoglucanase E
		PDE_01959	Alpha-mannosidase
		PDE_02102	Swollenin
		PDE_02648	Alpha 1,3-glucosidase
		*PDE_07945*	*Exoglucanase/cellobiohydrolyase Cel7A/CBHI*
		PDE_09279	Glycosyl hydrolase family 43 protein
		*PDE_09417*	*Glucoamylase Amy15A*
GO:0004553	Hydrolase activity, hydrolyzing O-glycosyl compounds	*PDE_01201*	*Alpha-amylase Amy13A*
		PDE_01302	Endoglucanase E
		PDE_01959	Alpha-mannosidase
		PDE_02004	Endo-1,6-beta-d-glucanase
		PDE_02102	Swollenin
		PDE_02648	Alpha 1,3-glucosidase
		*PDE_02736*	*Beta-glucosidase Cel3A/BGL1*
		PDE_04393	Amidohydrolase
		*PDE_07945*	*Exoglucanase/cellobiohydrolyase Cel7A/CBHI*
		PDE_09279	Glycosyl hydrolase family 43 protein
		*PDE_09417*	*Glucoamylase Amy15A*

The genes in italics indicate the genes among the top 10 extracellular glycoside hydrolase encoding genes

### Overexpression of *hepA* activated cellulolytic enzyme synthesis remarkably

**Fig. 4:**
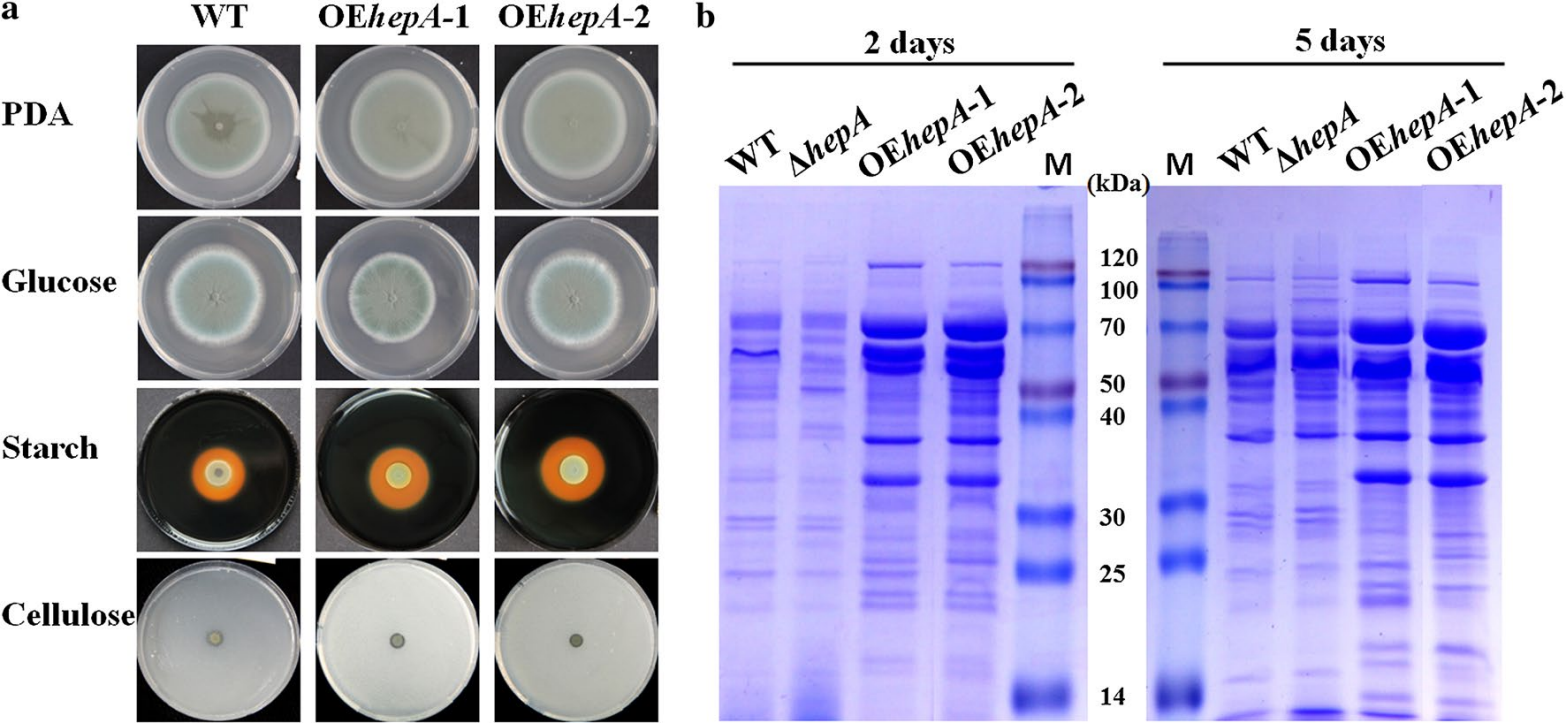
Colony morphology and SDS-PAGE analysis of WT and *hepA* overexpression (OE*hepA*) strains. **a** Colony morphology of 5-day-old cultures for WT and two *hepA* overexpression strains on PDA or VMM with 2 % glucose, 2 % starch, and 2 % cellulose at 30 °C. The 1 μL conidia solution of WT or OE*hepA* was dropped onto the agar at a density of 10^6^ conidia mL^−1^. **b** SDS-PAGE analysis of extracellular protein for WT, Δ*hepA*, and OE*hepA*

We doubted the ability of the *hepA* overexpression to activate the cellulase synthesis because the prominent extracellular cellulolytic enzyme gene expression was downregulated in Δ*hepA*, and HP1 has consistently been reported with dose-dependent effect on gene expression [34]. Two overexpression strains (OE*hepA*-1 and OE*hepA*-2, *hepA* overexpressed under *gpdA* strong constitutive promoter), which were selected randomly, indicated clearer hydrolysis halos than that of WT (Fig. 4a) when the strains were grown on VMM + starch or VMM + cellulose agar, suggesting higher amylase or cellulolytic enzyme activity in OE*hepA*.

Pure cellulose was used as the sole carbon source for the agar. We cultivated the WT, Δ*hepA*, and OE*hepA* in a submerged medium with wheat bran and cellulose because complex carbon sources from plant materials are more efficient than pure cellulose in promoting the expression of cellulolytic enzyme. The supernatants from the WT and mutants were profiled by SDS–PAGE (Fig. 4b). Equal volumes of supernatants were loaded. Significantly fainter protein bands were detected in Δ*hepA* than in WT, whereas darker protein bands were detected in OE*hepA*, especially in the range of 40–116 kDa, which is an area for the aggregation of glycoside hydrolases in particular. Most prominent glucoamylases, cellobiohydrolases, endoglucanases, and β-glucosidases were located in the area according to previous reports [33].

**Fig. 5:**
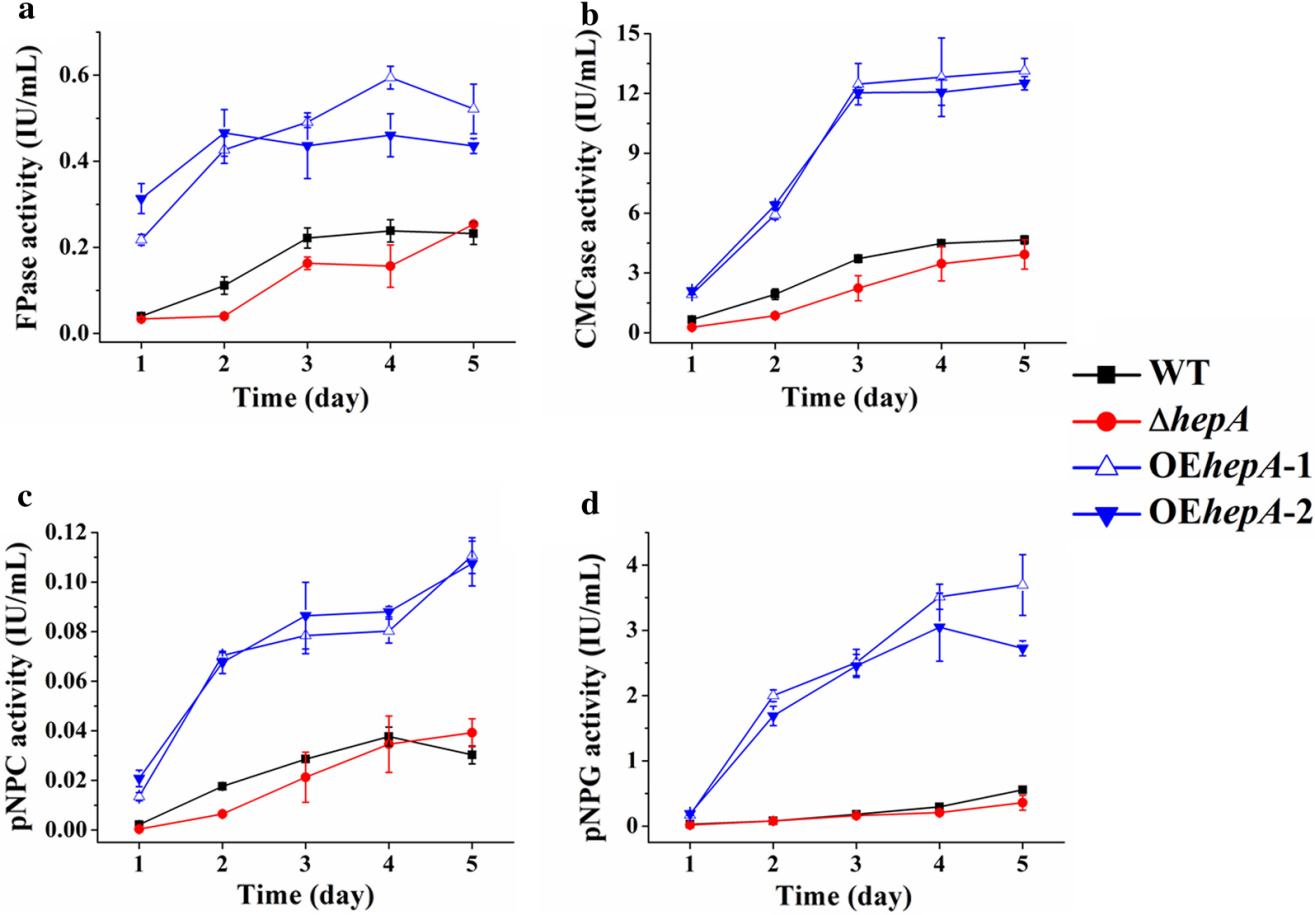
Cellulolytic activity assay of WT and various mutants. The strains were cultivated in liquid VMM supplemented with 1 % wheat bran and 1 % microcrystalline cellulose as carbon resources to induce cellulolytic enzyme gene expression. The strains were cultivated at 30 °C for 5 days. **a** Filter paper activity (FPA) represents the overall cellulase activity and combined activities of both endo- and exo-type cellulases. **b** CMcase activity represents the activity of endo-type cellulases. **c** p-nitrophenol-d-cellobioside (pNPC) activity represents the activity of exo-type cellulases. **d** p-nitrophenyl-d-glucopyranoside (pNPG) activity represents the activity of β-glucosidase. The values show the mean of three biological replicates, and the *error bar* indicates the standard deviation

Then, the levels of FPA (filter paper activity, representing overall cellulase activity), CMCase activity (representing endoglucanase activity), pNPC activity (representing cellobiohydrolyase activity), and pNPG activity (representing extracellular β-glucosidase activity) were assayed (Fig. 5). In the absence of HepA, FPA, CMCase, pNPC, and pNPG activities of Δ*hepA* decreased compared with that of the WT. On the fourth day, FPA, CMCase, pNPC, and pNPG of Δ*hepA* were 65.6, 77.3, 91.8, and 70.1 %, respectively. In contrast, both *hepA* overexpression strains (OE*hepA*-1 and OE*hepA*-2) showed significantly increased FPA, CMCase, pNPC, and pNPG activities compared with WT. For example, the FPA, CMCase, pNPC, and pNPG activities of OE*hepA*-1 on the fourth day were 2.5-, 2.9-, 2.2-, and 11.9-fold that of the WT, respectively. The introduction of a WT copy of *hepA* (R*PolaeA*) restored the cellulolytic enzyme synthesis defects of the Δ*hepA* mutant (Additional file 4: Fig. S3).

Were the changes in cellulolytic enzyme synthesis in the mutants caused by their different biomass levels? Performing an assay of the mycelia biomass according to the dry cell weight can be difficult, because wheat bran and cellulose were added in the fermentation medium as inducer and unconsumed wheat bran and cellulose interfered with the results. Thus, total intracellular protein concentration was determined to indicate the biomass. Except for some differences at the early state of cultivation (4 h), identical intracellular protein concentrations were observed in WT, Δ*hepA*, OE*hepA*, R*PohepA*, and R*AnhepA* after 24 h of cultivation, indicating that the reduced cellulolytic activities in Δ*hepA* or increased cellulolytic enzyme activities in OE*hepA* were not correlated with the biomass level (Additional file 5: Fig. S4).

### Cellulolytic enzyme gene expression and chromatin structures are regulated by the HepA

**Fig. 6:**
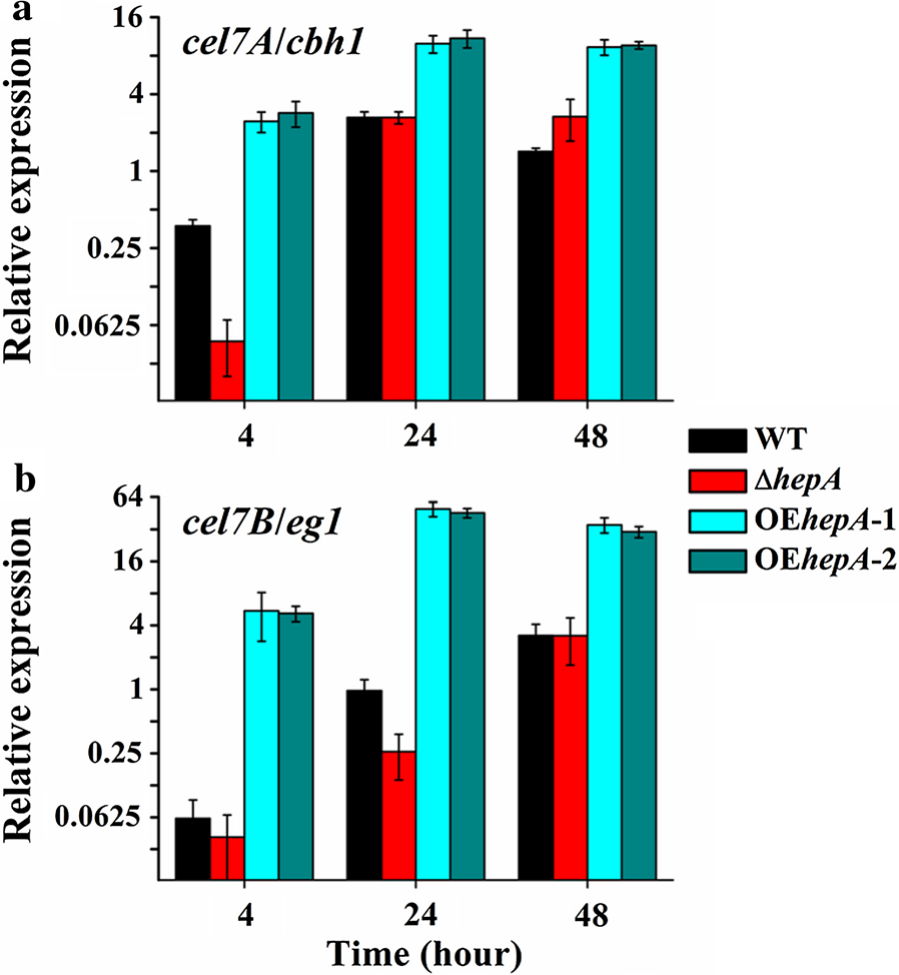
Expression levels of prominent cellulolytic enzyme genes determined using real-time quantitative PCR (qPCR). **a** Cellobiohydrolase gene *cel7A*/*chb1*. **b** Endoglucanase gene *cel7B*/*eg1*

A quantitative PCR was carried out to compare the transcription patterns of the cellulase genes, including the prominent cellobiohydrolase gene *cel7A*/*cbh1* and endoglucanase gene *cel7B*/*eg1* (Fig. 6). The transcript levels of *cel7A*/*cbh1* and *cel7B*/*eg1* in both *hepA* overexpression strains obviously and significantly increased. For OE*hepA*-1, the transcript level of *cel7A*/*cbh1* increased by 3.8- and 6.5-folds at 24 and 48 h compared with the WT, respectively (Fig. 6a). The transcript level of *cel7B*/*eg1* increased by 51.3- and 11.0-folds at 24 and 48 h compared with the WT, respectively (Fig. 6b). The results suggested that overexpression of *hepA* significantly activated the cellulolytic enzyme gene expression.

**Fig. 7:**
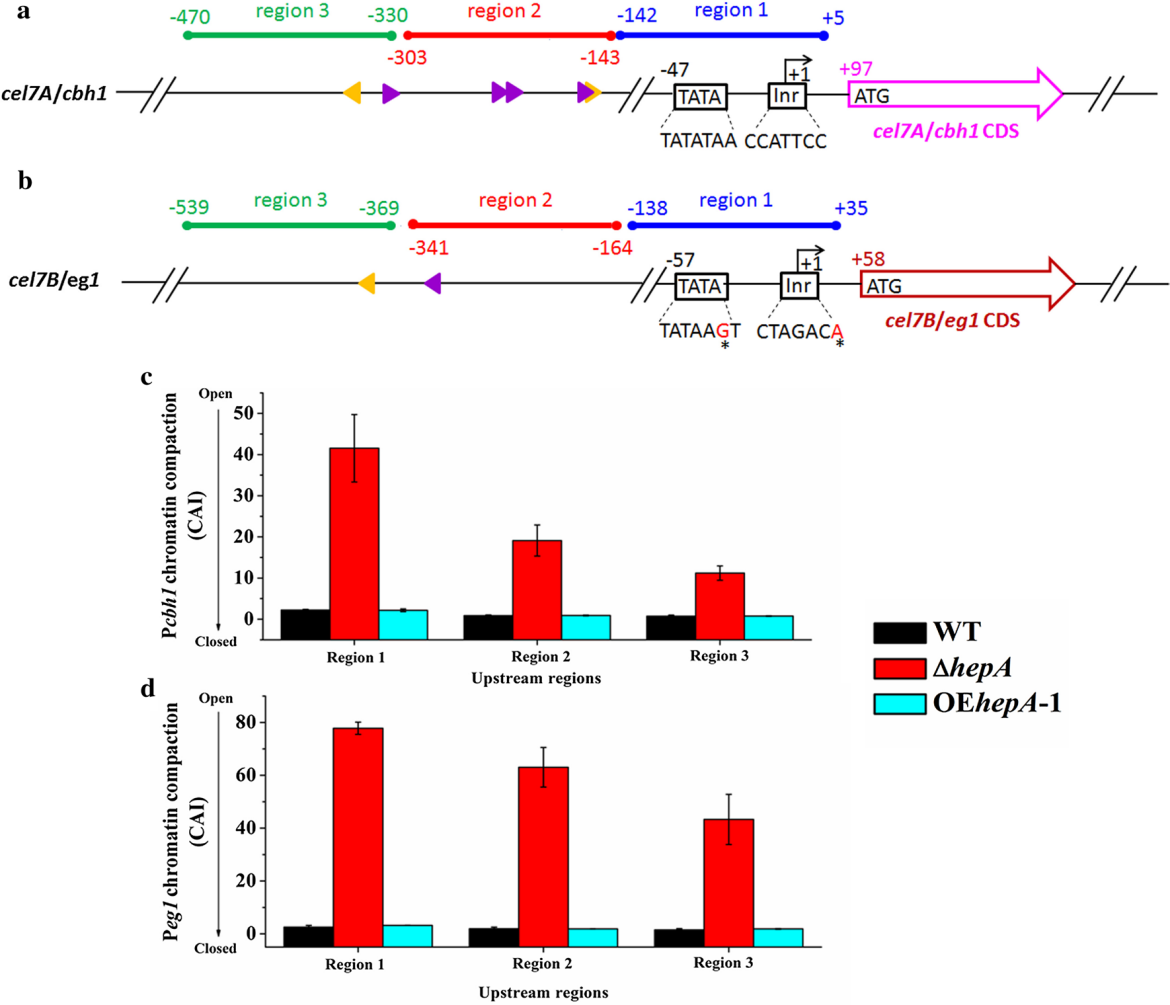
Strategy and results of CHART-PCR. **a**, **b** Overview on the upstream sequence and core promoters of *cel7A*/*chb1* and *cel7B*/*eg1*. The transcription start site is designated as +1. The initiator (Inr) and TATA elements (or box) were illustrated. The three chromatin regions investigated by CHART-PCR are indicated by *blue*, *red*, and *green bars*, respectively. For *cel7A*/*chb1*, region 1 covers from +5 to −142; region 2 covers from −143 to −303; region 3 covers from −330 to −470. For *cel7B*/*eg1*, region 1 covers from +35 to −138; region 2 covers from −164 to −341; region 3 covers from −369 to −539. The putative DNA-binding sites of CreA and XlnR are indicated by *orange* and *purple triangles*, respectively. The orientation of the *triangle* represents the orientation of the bind motif. Inr, initiator element; TATA, TATAbox. **c** CHART analysis of *cel7A*/*chb1*. **d** CHART analysis of *cel7B*/*eg1*. All values are means from measurements in triplicates and three biological experiments. The *error bars* indicate standard deviations

Transcriptional activation in eukaryotes is often accompanied by alterations in the chromatin structure at specific regulatory sites. We used CHART-PCR analysis to better comprehend the contribution of HepA to the changes in chromatin packing, especially the chromatin status of the core promoter and upstream region of cellulolytic enzyme gene. We first analyzed the upstream sequence and core promoters for *cel7A*/*cbh1* and *cel7B*/*eg1*. Eukaryotic core promoter refers to the minimal set of sequence elements required for accurate transcription initiation by the Pol II machinery. The initiator element (Inr) is the most common element found in combination with both TATA element (or box) and downstream promoter element (DPE) [35]. The Inr (CCATTCC) and TATA box (TATATAA) were found in the *cel7A* Pol II core promoter region (Fig. 7a), fitting with the consensus sequence of Inr (C/T)_2_AN(T/A)(C/T)_2_ and TATA box TATA(A/T)A(A/T). The Inr (CTAGACA) and TATA element (TATAAGT) were found in the *cel7B* Pol II core promoter region (Fig. 7b), with base variation (indicated by the asterisk) compared with the consensus sequence of Inr and TATA box. Both *cel7A* and *cel7B* bore the putative CreA-binding sites “5′-GGC(T/A)_3_-3′” [8] and putative XlnR-binding sites “5′-SYGGRG-3′” [36]. DPE elements were not found in both *cel7A* and *cel7B*. Three regions were designed for the CHART-PCR of *cel7A*/*cbh1* and *cel7B*/*eg1*. In the *cel7A*/*cbh1* upstream region, region 1 (+5 to −142), region 2 (−143 to −303), and region 3 (−330 to −470), were designed, respectively. In the *cel7B*/*eg1* upstream region, region 1 (+35 to −138), region 2 (−164 to −341), and region 3 (−369 to −539), were designed, respectively. Region 1 covered the core promoters region; Regions 2 and 3 were upstream the core promoters, which were considered for transcriptional regulatory protein (i.e., activator XlnR or repressor CreA) binding (Fig. 7a, b).

Next, we analyzed the chromatin status change mediated by HepA. In the Δ*hepA* mutant, the chromatin of all three tested upstream regions opened specifically in the cases of *cel7A*/*cbh1* and *cel7B*/*eg1*. The chromatin accessibility index increased remarkably (Fig. 7c, d). The results suggested that HepA was required for chromatin condensation, but this action was not essential for the repression of transcription because the opening of chromatin in the absence of HepA did not occur with the increase of gene expression (Fig. 6). Meanwhile, transcripts can even be detected at high levels in the *hepA* overexpression strain although the chromatin status of OE*hepA* was identical with that of WT. Chromatin closing or opening alone does not repress or activate gene expression. Among the three target regions for either *cel7A*/*cbh1* or *cel7B*/*eg1* in the Δ*hepA* mutant, region 1 showed the most relaxed chromatin status. The farther the regions were from the core promoters, the lower the opening degree of chromatin. Considering that region 1 covered the core promoter region, the result was expected because the disruption of the chromatin structure from the core promoter region or the proximal promoter region of a gene is a prerequisite to transcription initiation and elongation [37].

### Overexpression of *hepA* improved cellulase activity in the industrial strain RE-10

**Fig. 8:**
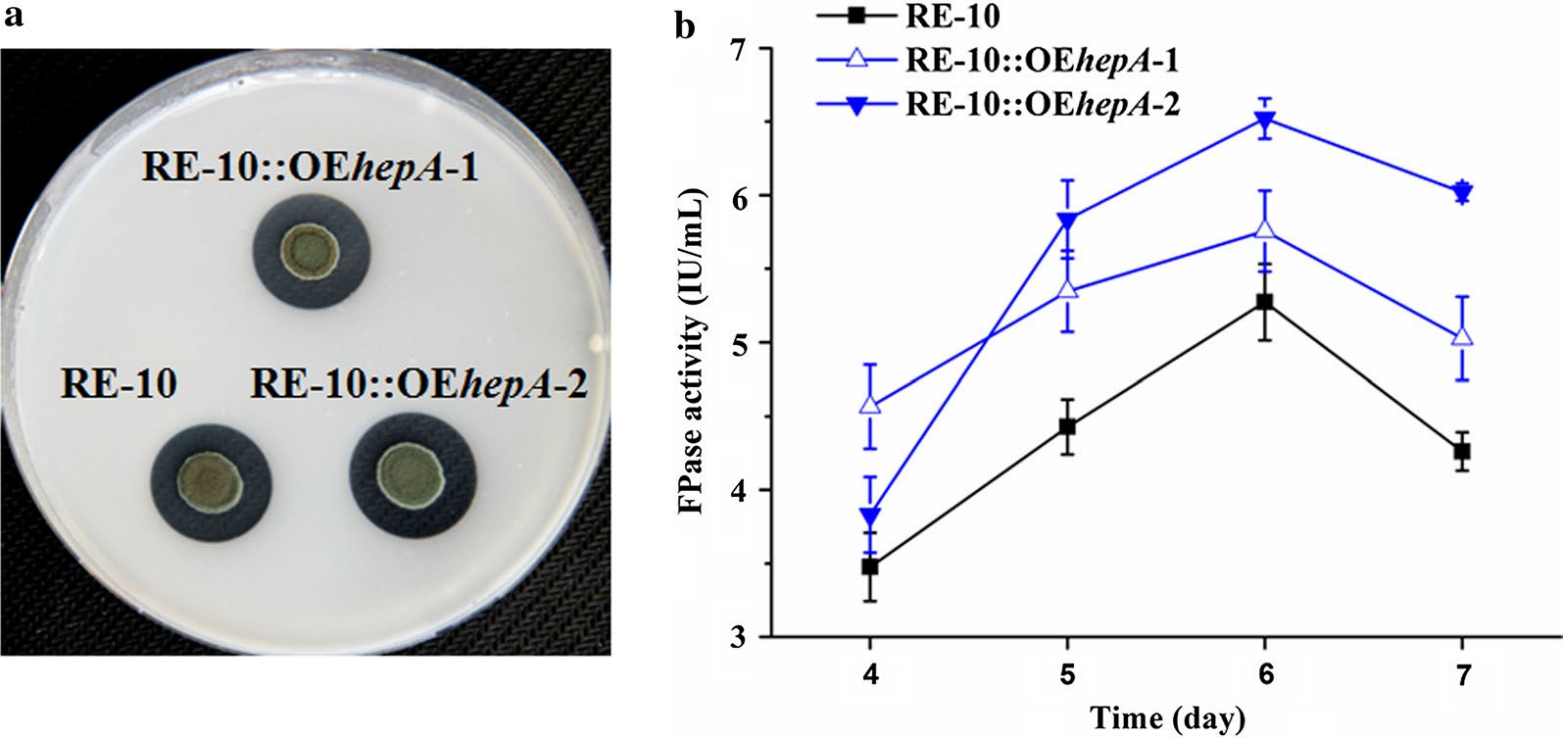
Cellulolytic activity assay of RE-10 and *hepA* overexpression strain in RE-10. **a** Colony morphology of 5-day-old cultures for RE-10 and two *hepA* overexpression strains (RE-10::OE*hepA*-1 and RE-10::OE*hepA*-2) on VMM with 2 % cellulose at 30 °C. **b** FPA of WT, RE-10::OE*hepA*-1, and RE-10::OE*hepA*-2

*P. oxalicum* has been used in the industrial production of cellulase. We overexpressed the *hepA* gene in the industrial strain RE-10 to enhance the cellulolytic enzyme synthesis of the *P. oxalicum* further. RE-10, with an improved 20-fold filter paper activity compared to *P. oxalicum* WT 114-2, included three genetic modifications: deletion of transcription repressor CreA, overexpression of transcription activator ClrB, and deletion intracellular β-glucosidase Bgl2 [26]. The overexpression of *hepA* can improve cellulase activity in the industrial strain RE-10. Both overexpression strains (RE-10::OE*hepA*-1 and RE-10::OE*hepA*-2), which were selected randomly, showed improved cellulase activity than RE-10. After 5 days of cultivation, the filter paper activities of RE-10::OE*hepA*-1 and RE-10::OE*hepA*-2 were 5.34 and 5.83 IU/mL, which increased by 20.8 and 31.9 % compared with that of RE-10, respectively (Fig. 8).

**Fig. 9:**
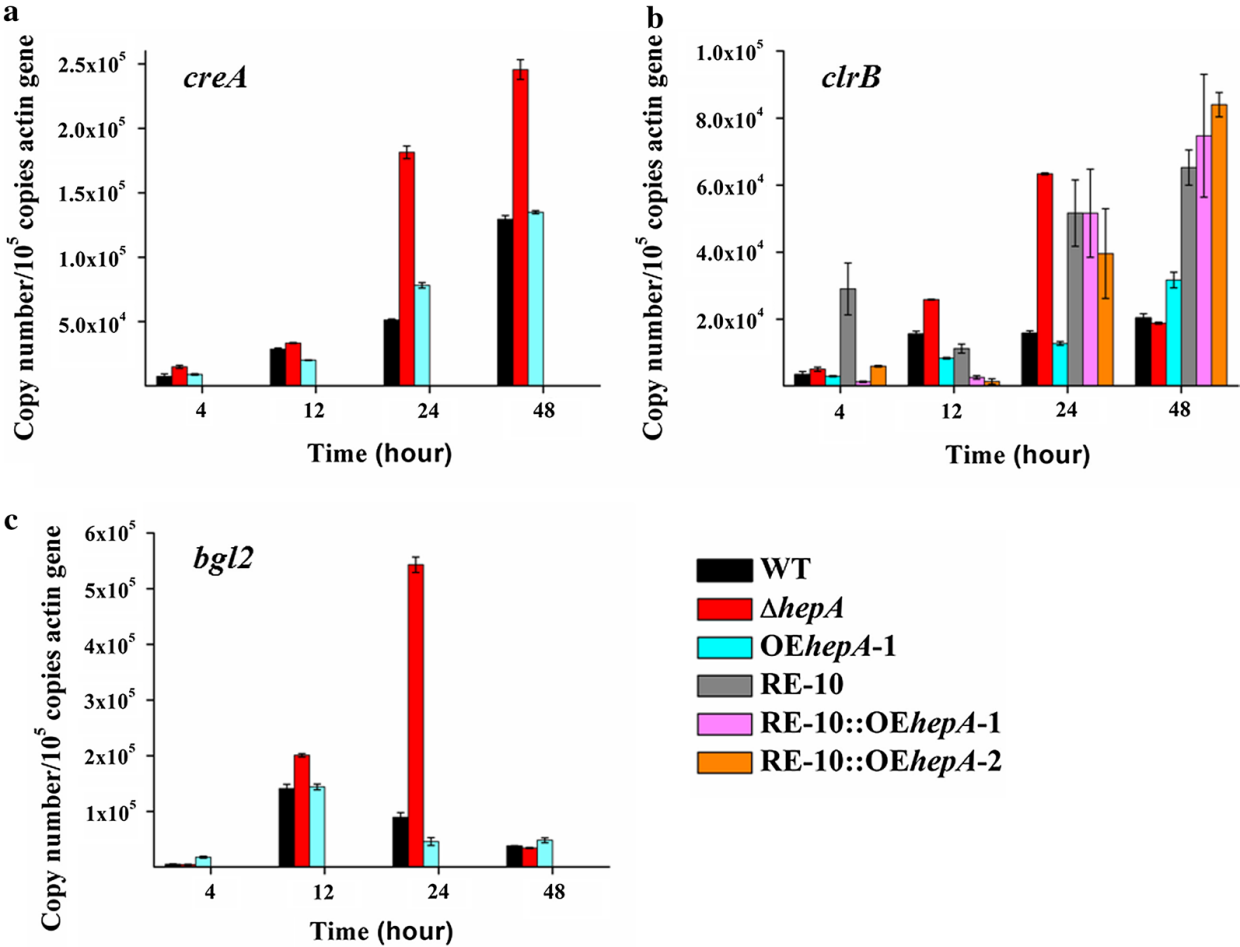
Expression levels of the *creA*, *clrB*, and *bgl2* in WT and different mutants determined using qPCR. **a** *creA*, **b** *clrB*, and **c** *bgl2*. There is no data for *creA* and *bgl2* expression in RE-10, RE-10::OE*hepA*-1, or RE-10::OE*hepA*-2, as both *creA* and *bgl2* were deleted in RE-10

Considering that transcription repressor CreA [5], transcription activator ClrB [38], and intracellular β-glucosidase Bgl2 [39] are target modification genes in RE-10, and thus far the most important regulators reported in *P. oxalicum*, their expression patterns were determined in WT, *hepA* deletion strain (Δ*hepA*), *hepA* overexpression strain (OE*hepA*), RE-10, and *hepA* overexpression in RE-10 strain (RE-10::OE*hepA)* (Fig. 9). All three genes were upregulated remarkably in Δ*hepA*; the *creA*, *clrB*, and *bgl2* expressions in Δ*hepA* were 3.5-, 4.0-, and 6.1-folds at 24 h, respectively. However, no difference of the *creA*, *clrB*, or *bgl2* expression was observed between WT and OE*hepA*, and no difference of the *clrB* expression was observed between RE-10 and RE-10::OE*hepA*.

## Discussion

According to our initial speculation, HepA might be a negative regulator for cellulolytic enzyme gene expression, as HP1 binding have always been implicated in heterochromatin formation and gene repression, whereas its loss leads to derepression of gene expression [20]. Therefore, we observed rather surprisingly that HepA is actually a positive regulator for the cellulolytic enzyme gene expression because the deletion of *hepA* showed the repression of cellulolytic enzyme gene expression, whereas the overexpression of *hepA* indicated an obvious activation of cellulolytic enzyme gene expression.

Indeed, in addition to the prominent roles of HP1 in gene repression, increasing evidence supports the view that HP1 proteins play a positive role in transcription. HP1 is not only required for the proper expression of genes that reside within the heterochromatin, but is also required for the expression of several euchromatic genes [40]. For example, many genes, such as *cdc2*, located in euchromatin are downregulated in the HP1 mutant *Drosophila* [41]. Reduced HP1 dosage leads to lower level of heat shock gene mRNA, whereas HP1 overexpression leads to increased *hsp70* mRNA [42]. However, no report has been made on the positive roles of HP1 in filamentous fungi. The involvement of *N. crassa* Hpo in heterochromatin formation, DNA methylation, and gene silencing has been reported [43]. The deletion of *hepA* in *A. nidulans* upregulated the SM gene expression; HepA occupancy at the ST cluster decreased during the transcriptional activation [22] and was required for transgene silencing [44]. Our results also showed that the vast majority (95.0 %) of genes that exhibited significant differences between Δ*hepA* and WT were upregulated in Δ*hepA* compared with those in WT (Fig. 3), suggesting that HepA played a main negative role in transcription. The positive regulation of the cellulolytic enzyme gene expression by HepA was an unexpected finding.

Although numerous studies have been conducted to elucidate the mechanism of the transcriptional regulation of cellulolytic enzyme genes by transcription regulatory proteins, few studies have been conducted to investigate the effect of chromatin status on their expressions. In the generally accepted model, transcriptionally repressed chromatin is condensed and transcriptionally active chromatin is decondensed. For example, in *T. reesei* strain bearing a *xyr1* deletion, the sophorose-mediated inductions of the two prominent cellulase-encoding genes *cbh1* and *cbh2* expressions are lost and the degrees of chromatin opening are strongly reduced [24]. CreI was also reported to play an essential role in correct nucleosome positioning within the main cellulase gene *cbh1* promoter [45].

The interaction between HP1 and chromatin was speculated to be static; HP1 acted as “molecular glue” locking nucleosomes together tightly, triggering a repressive chromatin structure once it is targeted to specific promoters, thereby blocking the access of the transcription machinery or activators to the gene [46]. However, our results on the prominent cellulase gene *cbh1* and *eg1* expressions and the results of CHART-PCR did not fit the classical view of chromatin structures and gene expression. In particular, we found that the deletion of *hepA* led to highly opened chromatin, without increase for cellulolytic enzyme gene expression. The overexpression of *hepA* led to the active cellulolytic enzyme gene but without chromatin structure modification. These results suggest that chromatin condensation or decondensation in the promoter region alone does not repress or activate gene expression. The opening of chromatin was not always positively correlated with gene expression, but showed considerable variety in different genes and cultures. In *T. reesei* QM6a-CreI_96_ (a truncated CreI having left a sequence that would only encode for one of the two zinc finger regions of CreI, 96 aa long), the *cbh1* and *cbh2* gene expressions increased with simultaneous opening of chromatin during the strain on d-Glucose. No direct correlation between gene expression and chromatin accessibility was observed in the QM6a-ΔCreI strain, and no correlation of gene expression was observed in either QM6a-CreI_96_ or QM6a-ΔCreI with the simultaneous opening of chromatin in the strains during sophorose induction [47]. Some studies have shown that the repression or silencing of genes is not caused by the physical inaccessibility of condensed chromatin domains to transcription factors; compact chromatin domains are readily accessible to large macromolecules, such as protein and dextrans [48]. The silent chromatin in *S. pombe* also exhibits high MNaseI sensitivity [49]; chromatin condensation is independent of HP1 homologue Swi6, although most gene silencing disappear at the subtelomeric region in the Δ*swi6* mutant [50]. Interestingly, GO analysis showed downregulation of extracellular cellulolytic enzyme genes, along with upregulation of genes involved in protein synthesis and downregulation of one secondary metabolic gene cluster. The research by Arvas showed that *T. reesei* genes involved in major biosynthetic activities such as protein synthesis and energy-related genes, were negatively correlated with extracellular specific protein production rate (SPPR), while genes of secondary metabolism were positively correlated with SPPR [51]. So, the decreased cellulase activity in Δ*hep*A might be along with possible derepression of translation machinery the same as described by Arvas et al.

Although the overexpression of *hepA* in WT could lead to two to threefold improvement of PFA, the overexpression of *hepA* in the industry strain RE-10 only brought 20–30 % improvement of PFA. RE-10 is a genetic modification strain bearing the deletion of transcription repressor CreA, overexpression of transcription activator ClrB, and deletion of intracellular β-glucosidase Bgl2 [26]. Although CreA/Cre1 and ClrB/Clr2 are prominent for their functions in cellulolytic enzyme gene expression regulation [6, 7], their regulons are not limited to cellulolytic enzyme genes. *T. reesei* Cre1 plays a major role in the regulation of 250 genes, including cellulolytic enzyme genes and genes linked to transcription regulation (e.g., the helicase SNF2 involved in chromatin remodeling and the transcriptional regulator MedA involved in sporulation) [13]. *P. oxalicum* CreA and ClrB were reported to be involved in the regulation of at least 1100 and 220 genes, respectively. Furthermore, the simultaneous overexpression of ClrB and lack of CreA caused stronger synergistic effect on gene expression, affecting more than 2000 genes [38], including 429 genes, which were also detected in the regulon of *hepA* deletion strain (data not shown). These genes not only included cellulolytic enzyme gene, but also some transcription factors encoding genes, such as the *brlA* (PDE_00087) involved in both sporulation and cellulase formation [52], and Rho GTPase encoding gene *rhoC1* (PDE_03690) involved in G-protein-mediated signal transduction and associated with hemicellulose degradation [53, 54]. Some genes, which are attributed to the improvement of cellulolytic enzyme gene expression regulated in OE*hepA*, overlapped with the genes regulated in RE-10. This overlapping might be the reason the overexpression of *hepA* in RE-10 did not lead to so significant improvement like that of the overexpression of *hepA* in WT.

HepA is obviously involved in cellulolytic enzyme gene expression, and HepA could be a promising target for genetic modification to improve cellulolytic enzyme synthesis. However, the mechanisms of HepA-mediated cellulolytic enzyme gene activation are not fully understood. No difference of the *creA*, *clrB*, or *bgl2* expression was observed between WT and OE*hepA*, suggesting that the activation of the cellulolytic enzyme gene expression in the *hepA* overexpression strain was not attributed to the above three regulators. In *A. nidulans*, HepA and histone methyltransferase Clr4 form protein complex involving in gene repression [22]. Interestingly, in the screen for *P. oxalicum* LaeA (a putative histone methyltransferase) interaction protein in yeast two-hybrid library, HepA was one of the screened proteins that interacted with LaeA (self-communication, data unpublished). LaeA, as a positive regulator of cellulolytic enzyme gene [23], can prevent HepA binding and the formation of repressive chromatin [22]. LaeA’s partner VeA is also involved in cellulase gene expression [55]. Whether HepA can interact with LaeA (just like Clr4) and form the complex in vivo? Therefore, additional genetic analysis of the integration of HepA and LaeA, and additional biochemical analysis of HepA-containing complexes could provide insights into its role in cellulolytic enzyme gene activation.

## Conclusions

The results obtained in this study indicate that HepA is required for chromatin condensation of prominent cellulase genes. However, the opening of chromatin mediated by the deletion of *hepA* was not positively correlated with cellulase gene activation. The activation of cellulase gene caused by *hepA* overexpression did not go along with the chromatin status modification. The results suggested that chromatin condensation or decondensation alone in the promoter region does not repress or activate gene expression. HepA is a positive regulator for cellulolytic enzyme gene expression and could be a promising target for genetic modification to improve cellulolytic enzyme synthesis.

## Methods

### Fungal strains, mediums, and culture conditions

*Penicillium oxalicum* WT 114-2 (CGMCC 5302), RE-10 [26], and mutants were cultivated on wheat bran extract agar slants for 5 days at 30 °C. For phenotypic analysis, we selected the potato dextrose agar (PDA) medium and Vogel’s minimal medium (VMM) added with different carbon resources (2 % glucose, 1 % microcrystalline cellulose, and 1 % soluble starch). A 1 μL conidia suspension was spotted onto the medium and cultivated at 30 °C for 5 days.

### Genetic manipulations

We constructed deletion and overexpression cassettes with double-joint PCR [56]. WT genomic DNA was used as the template of the 5′- and 3′-flanking regions of *hepA* gene (1767 and 1499 bp, respectively) with primer pairs DhepA-UF/DhepA-hph-UR and DhepA-hph-DF/DhepA-DR to construct the *hepA* deletion strain. The marker gene hygromycin B (*hph*) (2425 bp) was amplified from plasmid Psilent1 [57] using primer pairs hph-F/hph-R. The three PCR fragments were fused with fusion PCR. The products were then amplified using nest primer pairs DhepA-NF/DhepA-NR. The PCR products (4826 bp) were transformed into WT to obtain Δ*hepA*. We used primer pairs RhepA-F/RhepA-R to obtain intact *hepA* sequence (open reading frame, 5′-flanking regions including promoter region, and 3′-flanking regions including terminator sequence) 2219 bp and complement the Δ*hepA* mutant with the *P. oxalicum* WT *hepA*. The resistant gene pyrithiamine (*ptrA*) was amplified from plasmid pME2892 [58] using primer ptra-F/ptra-R. The two fragments were fused and amplified using nest primer RhepA-NF/RhepA-NR. The PCR products (4068 bp) were transformed into Δ*hepA* to obtain the complement strain R*PohepA*. We used the primer pairs ANhepA-F/ANhepA-R to obtain intact *hepA* sequence (open reading frame, 5′-flanking regions including promoter region, and 3′-flanking regions including terminator sequence) 2339 bp to complement the Δ*hepA* mutant with the *A*. *nidulans hepA*. The marker gene *ptrA* was also amplified from plasmid pME2892. The two PCR fragments were fused and amplified using nest primer ANhepA-NF/ANhepA-NR. The PCR products (4274 bp) were transformed into Δ*hepA* to obtain complement strain R*AnhepA*. To construct the *hepA* overexpression strain in WT, the primers OEhepA-F/OEhepA-R, gpdA-F/gpdA-R, and hph-F/hph-R were used to obtain *hepA* open reading frame and 3′-flanking regions, including terminator sequence (1346 bp), the *gpdA* (glyceraldehyde-3-phosphate dehydrogenase) promoter (1256 bp), and resistant gene hygromycin B (*hph*) (2425 bp), respectively. The three PCR fragments were fused and amplified using nest PCR primer OEhepA-NF/OEhepA-NR. The PCR products (4595 bp) were transformed directly into *P. oxallicum* 114-2 to obtain OE*hepA*. The promoter region of the gene PDE_02864, which encodes 40S ribosomal protein S8 [38], was amplified with primer pairs DF/DR (1872 bp) to construct the *hepA* overexpression strain in the industry strain *P*. *oxalicum* RE-10. The open reading frame of *hepA* was amplified with primer REhepA-F/REhepA-R. The fragments were fused and amplified using nest primer RE-NF/RE-DR. The PCR products (6482 bp) were transformed into RE-10 to obtain RE-10::OE*hepA*. All primers used are provided in the Additional file 6: Table S2.

The transformations constructed in this study were further confirmed by Southern blot using a DIG Easy Hyb kit (Roche Diagnostics, Germany) based on the manufacturer’s protocol. Genomics DNA was digested with the restriction of endonuclease and separated from 0.75 % agarose gel electrophoresis. The DNA was transferred to the Hybond-N + nylon membranes (Amersham Biosciences/GE Healthcare, USA). Primer DhepA-SF/DhepA-SR, OEhepA-SF/OEhepA-SR, RpohepA-SF/RpohepA-SR, and RanhepA-SF/RanhepA-SR were used to amplify the probe from the *P. oxallicum* 114-2 genomics DNA to verify the *hepA* deletion (Δ*hepA*), *hepA* overexpression (OE*hepA*)*, P. oxalicum hepA* complement (R*PohepA*), and *A. nidulans hepA* complement (R*AnhepA*) strains, respectively. The hybridization DNA sizes in WT and genetic manipulate strains were different. All primers used are provided in the Additional file 6: Table S2. The strategy and results of Southern blot are provided in the Additional file 7: Figure S5.

### Cellulolytic activity assay

For fermentation, VMM added glucose (2 %, w/v) was used for hyphal growth for 22 h at 30 °C. The mycelia were then harvested by vacuum filtration, and 0.3 g of mycelia was transferred into a 50 mL fermentation medium. We used the VMM added with 1 % wheat bran and 1 % microcrystalline cellulose as carbon for the WT, Δ*hepA*, OE*hepA*, R*PohepA*, and R*AnhepA* fermentations. For the RE-10 and RE-10::OE*hepA* strains, we used a high carbon source medium containing 3.6 % wheat bran, 3.6 % avicel, 2.1 % soybean meal, 0.5 % KH_2_PO_4_, and 0.05 % MgSO_4_ for fermentation. The fermented broths were collected by centrifugation to remove the medium. Filter paper activity (FPA), carboxymethyl cellulose activity (CMCase), p-nitrophenol-D-cellobioside activity (pNPCase), and p-nitrophenyl-D-glucopyranoside activity (pNPGase) were measured according to the methods described by Li [38]. Briefly, filter paper Whatman No. 1 (GE, England), CMC-Na (Sigma, USA), p-nitrophenol-D-cellobioside (pNPC) (Sigma, USA), and p-nitrophenyl b-D-glucopyranoside (pNPG) (Sigma, USA) were used as substrates. The enzyme reactions were performed in 0.2 M of NaAc–HAc buffer (pH 4.8) at 50 °C for 60, 30, 30, and 30 min, respectively. The reducing sugar was quantified via the DNS method. The enzyme reactions were performed in the buffer used above. One enzyme activity unit (U) was defined as the amount of enzyme necessary to generate 1 μmol of glucose or pNP per minute. Sodium dodecyl sulfate–polyacrylamide gel electrophoresis (SDS-PAGE) was then conducted. Supernatants (24 μL) were loaded in a 12 % polyacrylamide gel. Coomassie blue G-250 stain reagent was used for staining.

### Transcriptome assay and GO analysis

The culture method was similar to that described in the part of “cellulolytic activity assay.” After 48 h of cultivation, total RNA was extracted from the frozen mycelia after lyophilization using the RNAisoTM reagent (TaKaRa, Dalian, China) and incubated with 10 U DNase I (Takara, Dalian, China) for 30 min at 37 °C to remove genomic DNA. Transcriptome assay based on Illumina sequencing was performed by Realbio (Realbio, Shanghai, China). Sequenced reads were mapped against predicted transcripts from the *P. oxalicum* 114-2 genome using SOAP2 software for short oligonucleotide alignment (http://www.soap.genomics.org.cn/soapaligner.html). Transcript abundance (reads per Kb per million reads, RPKM) [59] genes with significantly different expression levels were identified through a significance test with combined thresholds (FDR ≤ 0.001 and fold change ≥2) [30]. Blast2GO was used for function enrichment analysis of the gene sets with threshold at FDR ≤ 0.05 [31].

### qRT-PCR analysis

The culture method was similar to that described in the part of “cellulolytic activity assay.” After 4, 24, and 48 h of cultivation, the total RNA was extracted using the Trizol reagent (TaKaRa, Japan) and cDNA synthesis was performed using the PrimeScript RT reagent Kit (TaKaRa, Japan). Primers CBH-QF/CBH-QR, EG-QF/EG-QR, creA-QF/creA-QR, clrB-QF/clrB-QR, and bgl2-QF/bgl2-QR were used to amplify *cel7A*/*cbh1*, *cel7B*/*eg1*, *creA*, *clrB*, and *bgl2*, respectively. Quantitative PCR was performed on Roche 480 LightCycler (Roche, Mannheim, Germany) using SYBR Premix Ex Taq™ (TaKaRa, Japan). Three biological replicates and two experiment replicates were required for one sample. Actin gene was used for data normalization. Primers used in the qRT-PCR analysis are listed in the Additional file 6: Table S2.

### Chromatin accessibility real-time PCR (CHART-PCR)

CHART-PCR assay was performed according to the methods described by Mello-de-Sousa [47]. Spores of WT, Δ*hepA* mutant, and OE*hepA* strains were cultivated on wheat bran extract agar slants at 30 °C for 5 days, and washed by 0.9 % NaCl with 0.1 % Tween 20. The spore suspension was transferred into a 1× Vogel’s medium with 2 % glucose as the carbon source for 22 h. The mycelia were harvested by vacuum filtration, and portions (0.3 g) were transferred into a 50 mL medium containing 1× Vogel’s medium with 1 % wheat bran and 1 % microcrystalline cellulose as carbon source. The samples from 24 h after induction were collected and ground in liquid nitrogen. Then, 100 mg of powder was pushed into 1 mL of nuclease digestion buffer (250 mM sucrose, 60 mM KCl, 15 mM NaCl, 0.05 mM CaCl_2_, 3 mM MgCl_2_, 0.5 mM DTT, and 15 mM pH 7.5 Tris–HCl). Subsequently, 10 μL RNase-free DNase I (TaKaRa Biotechnology) was added to 100 μL samples at 37 °C for 5 min. The reaction was stopped by adding 100 μL of termination buffer with 20 mM EDTA and 2 % SDS. The same amounts of phenol–chloroform and chloroform were added for protein extraction. The supernatant was treated with 2 μL 1 mg/mL RNaseA at 37 °C for 15 min. The DNA was precipitated with 0.3 M NaAc and two volumes of ethanol. The DNA was then suspended with 20 μL of double-distilled water. qPCR analysis of the DNase I-treatment samples was performed to measure the relative abundance of target regions. Primers CBH1-F2/CBH1-R2, CBH1-F3/CBH1-R3, and CBH1-F4/CBH1-R4 were used to amplify the *cel7A*/*cbh1* upstream region 1 (+5 to −142), region 2 (−143 to −303), and region 3 (−330 to −470), respectively. Primers EG1-F1/EG1-R1, EG1-F2/EG1-R2, and EG1-F3/EG1-R3 were used to amplify the *cel7B*/*eg1* upstream region 1 (+35 to −138), region 2 (−164 to −341), and region 3 (−369 to −539), respectively. qRT-PCR was performed using SYBR Premix Ex Taq (Perfect Real-time, TaKaRa Biotechnology) with a LightCycler 480 system and software Version 4.0 (Roche, Mannheim, Germany). Each sample was prepared in triplicate. The chromatin accessibility index (CAI) was defined as CAI = 1/Ds/Dc, where Ds is the amount of intact DNA detected for each target region and Dc is the amount of intact DNA detected for the promoter regions of housekeeping gene *sar1*. The amount of intact DNA was calculated by comparing the threshold values of PCR amplification plots with the standard curve generated for each primer set using serial dilutions of genomic, uncut DNA. All primers used are listed in the Additional file 6: Table S2.

## Additional files

10.1186/s13068-016-0624-9 Saturation analysis of digital gene expression profiling for WT and Δ*hepA*. (A) WT. (B) Δ*hepA*.
10.1186/s13068-016-0624-9 List of upregulated genes (≥twofold, FDR < 0.05) in ∆*hepA* compared with WT with significantly enriched GO terms (GO category: molecular function).
10.1186/s13068-016-0624-9 Gene expression analysis of the secondary metabolic gene cluster 15 in Δ*hepA* compared with WT. The copy number of unambiguous transcripts for each gene was normalized to RPKM (reads per kilobases per million reads). PDE_04008, putative HC-toxin efflux carrier; PDE_04009, uncharacterized protein; PDE_04010, uncharacterized protein; PDE_04011, uncharacterized protein; PDE_04012, putative transcription factor sre2; PDE_04013, uncharacterized protein; PDE_04014, uncharacterized ATP-dependent helicase; PDE_04015, isotrichodermin C-15 hydroxylase; PDE_04016, 7-alpha-hydroxycholest-4-en-3-one 12-alpha-hydroxylase; PDE_04017, lovastatin nonaketide synthase; PDE_04018, lovastatin nonaketide synthase; PDE_04019, uncharacterized protein; PDE_04020, zinc-type alcohol dehydrogenase-like protein; PDE_04021, uncharacterized protein; PDE_04022, RNA export protein; PDE_04023, uncharacterized protein; PDE_04024, putative branched-chain-amino-acid aminotransferase; PDE_04025, isotrichodermin C-15 hydroxylase; PDE_04026, uncharacterized protein; PDE_04027, 2,6-dihydropseudooxynicotine hydrolase; PDE_04028, protein TOXD; PDE_04029, uncharacterized protein. *: No transcriptome data.
10.1186/s13068-016-0624-9 Cellulolytic activity assay of WT and the recomplement strains.
10.1186/s13068-016-0624-9 Total intracellular protein concentration assay for WT and vavious mutants.
10.1186/s13068-016-0624-9 Primers used in this study.
10.1186/s13068-016-0624-9 Strategies and results of Southern blot for different mutants. (A) *hepA* deletion strain, Δ*hepA*. (B) *hepA* overexpression strain in WT, OE*hepA*. (C) *P. oxalicum hepA* recomplement strain, R*PohepA*, and *A. nidulans hepA* recomplement strain, R*AnhepA*. (D) *hepA* overexpression strain in RE-10, RE-10::OE*hepA*.

## Abbreviations

WT: wild type
FPA: filter paper activity
SDS–PAGE: sodium dodecyl sulfate polyacrylamide gel electrophoresis
CHART-PCR: chromatin accessibility real-time PCR
CAI: chromatin accessibility index
