# Supplementary figures and images for: Expression and chromatin structures of cellulolytic enzyme gene regulated by heterochromatin protein 1
Source: Biotechnol Biofuels. 2016 Oct 3;9:206. doi: 10.1186/s13068-016-0624-9 (PMC5048463; doi:10.1186/s13068-016-0624-9)

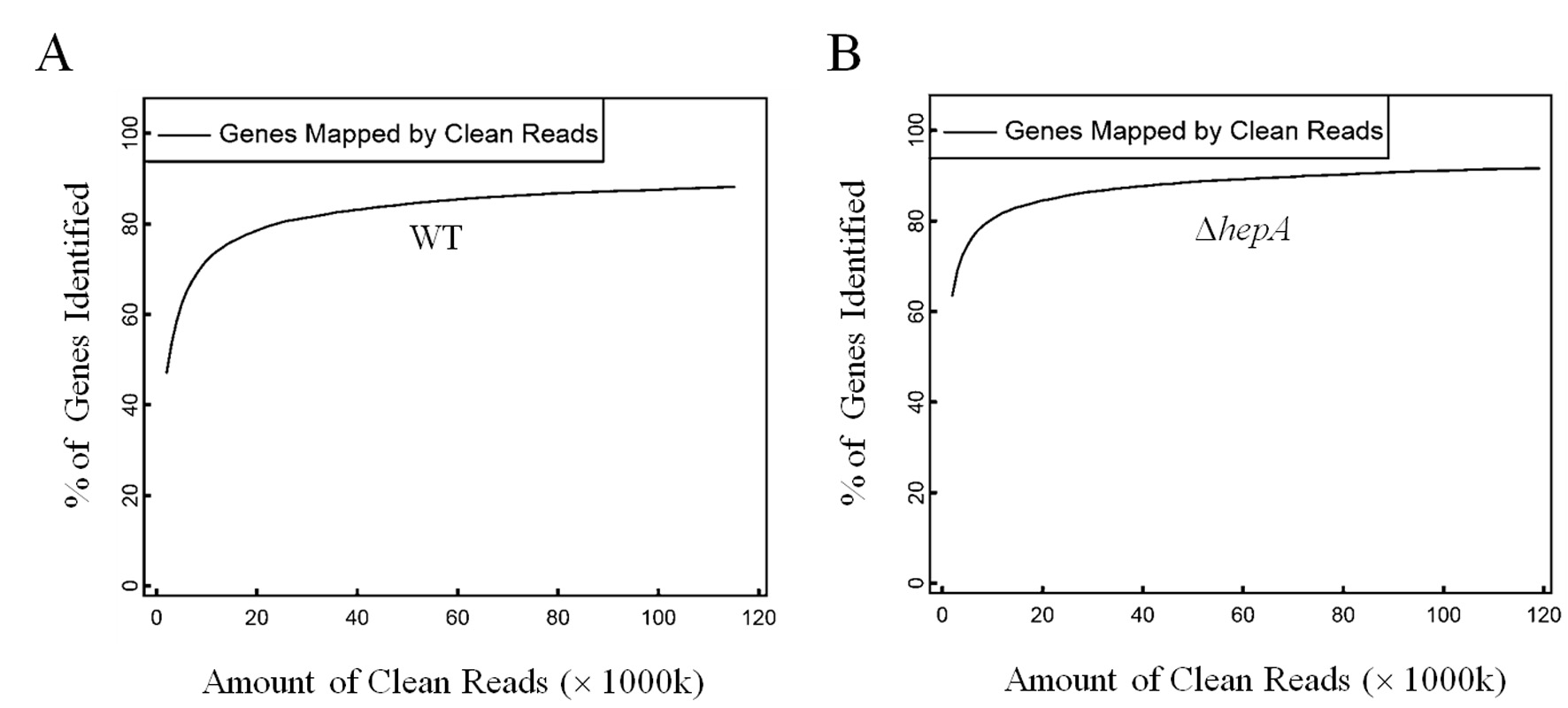

Supplement: Supplementary file 1 — 10.1186/s13068-016-0624-9 Saturation analysis of digital gene expression profiling for WT and ΔhepA. (A) WT. (B) ΔhepA. [file 13068_2016_624_MOESM1_ESM.jpg]

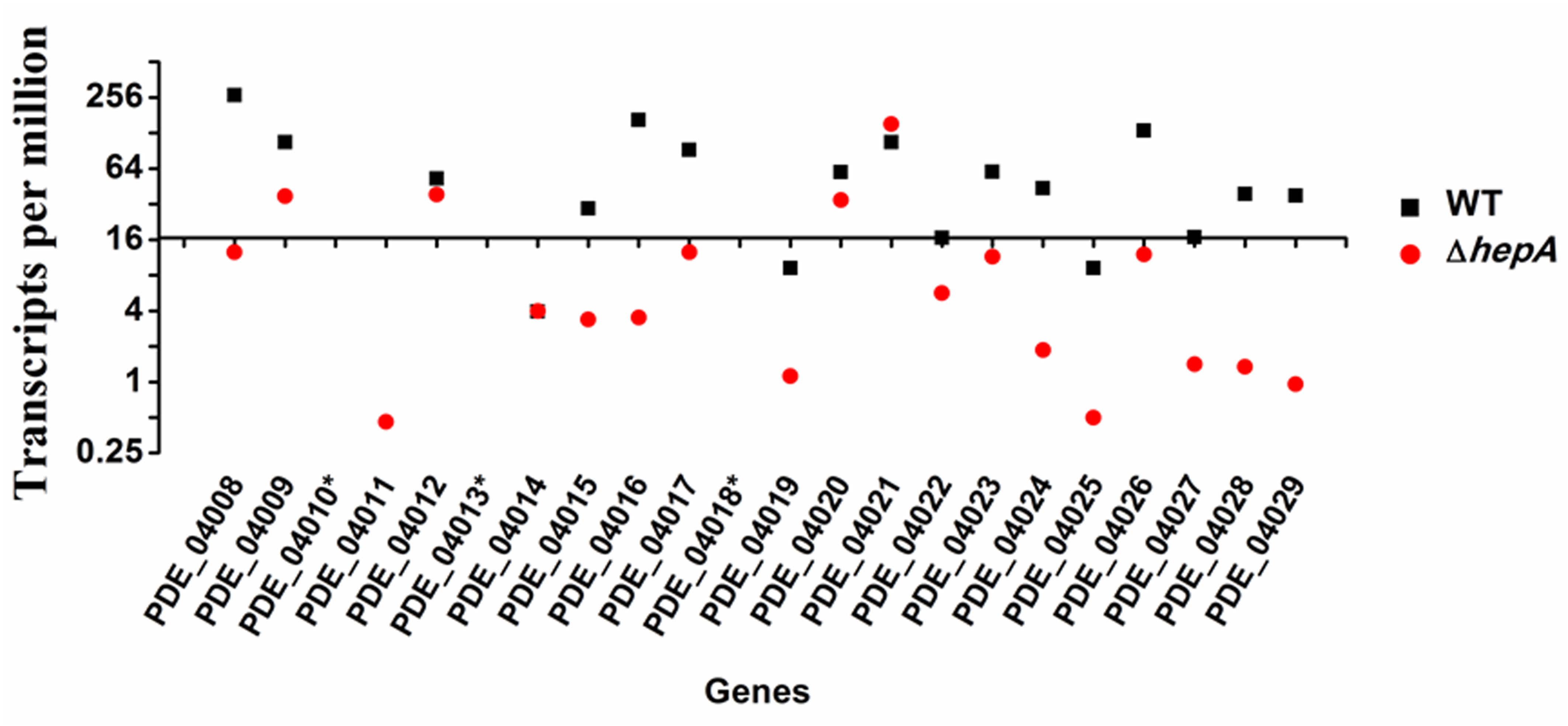

Supplement: Supplementary file 3 — 10.1186/s13068-016-0624-9 Gene expression analysis of the secondary metabolic gene cluster 15 in ΔhepA compared with WT. The copy number of unambiguous transcripts for each gene was normalized to RPKM (reads per kilobases per million reads). PDE_04008, putative HC-toxin efflux carrier; PDE_04009, uncharacterized protein; PDE_04010, uncharacterized protein; PDE_04011, uncharacterized protein; PDE_04012, putative transcription factor sre2; PDE_04013, uncharacterized protein; PDE_04014, uncharacterized ATP-dependent helicase; PDE_04015, isotrichodermin C-15 hydroxylase; PDE_04016, 7-alpha-hydroxycholest-4-en-3-one 12-alpha-hydroxylase; PDE_04017, lovastatin nonaketide synthase; PDE_04018, lovastatin nonaketide synthase; PDE_04019, uncharacterized protein; PDE_04020, zinc-type alcohol dehydrogenase-like protein; PDE_04021, uncharacterized protein; PDE_04022, RNA export protein; PDE_04023, uncharacterized protein; PDE_04024, putative branched-chain-amino-acid aminotransferase; PDE_04025, isotrichodermin C-15 hydroxylase; PDE_04026, uncharacterized protein; PDE_04027, 2,6-dihydropseudooxynicotine hydrolase; PDE_04028, protein TOXD; PDE_04029, uncharacterized protein. *: No transcriptome data. [file 13068_2016_624_MOESM3_ESM.jpg]

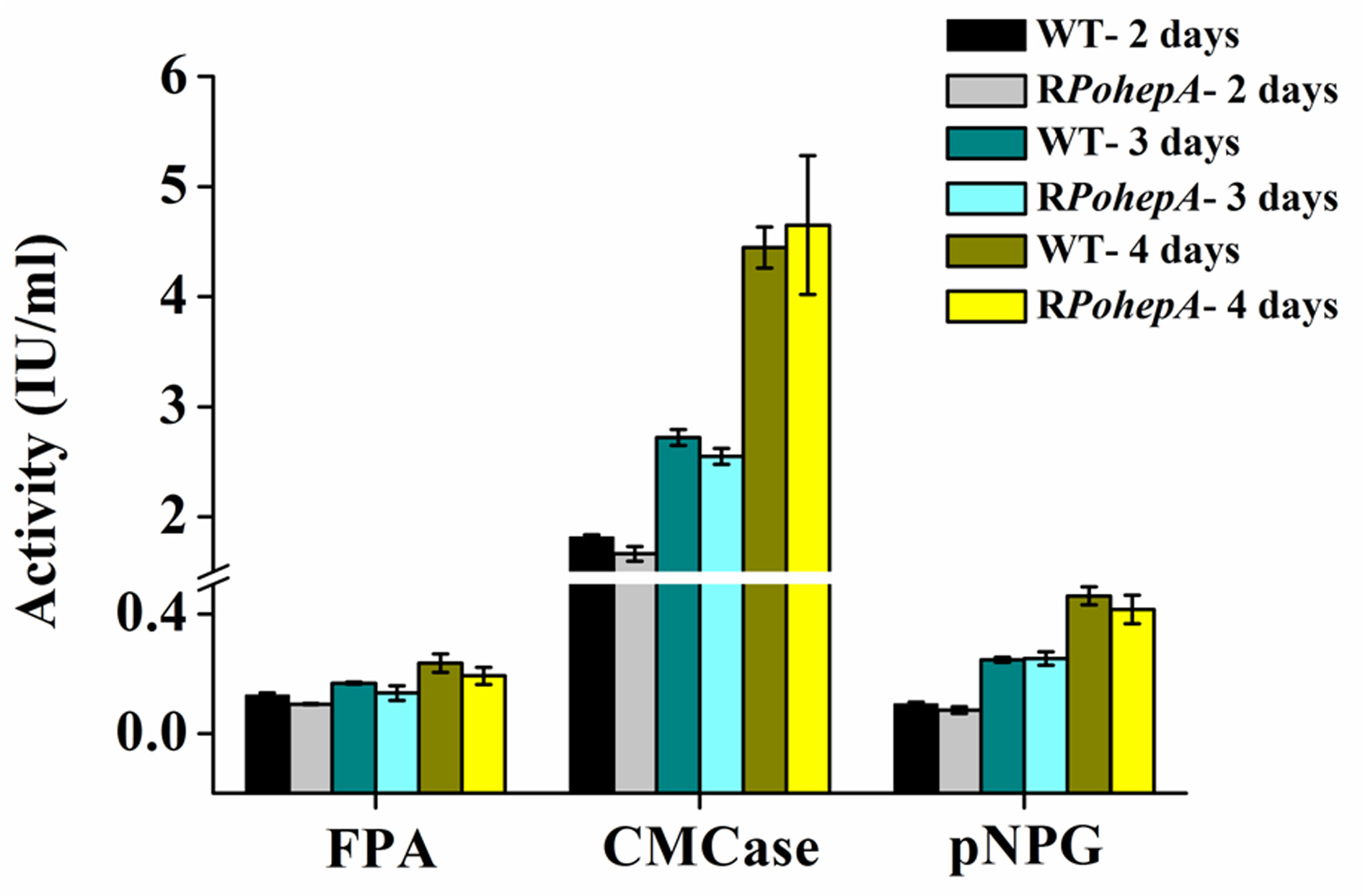

Supplement: Supplementary file 4 — 10.1186/s13068-016-0624-9 Cellulolytic activity assay of WT and the recomplement strains. [file 13068_2016_624_MOESM4_ESM.jpg]

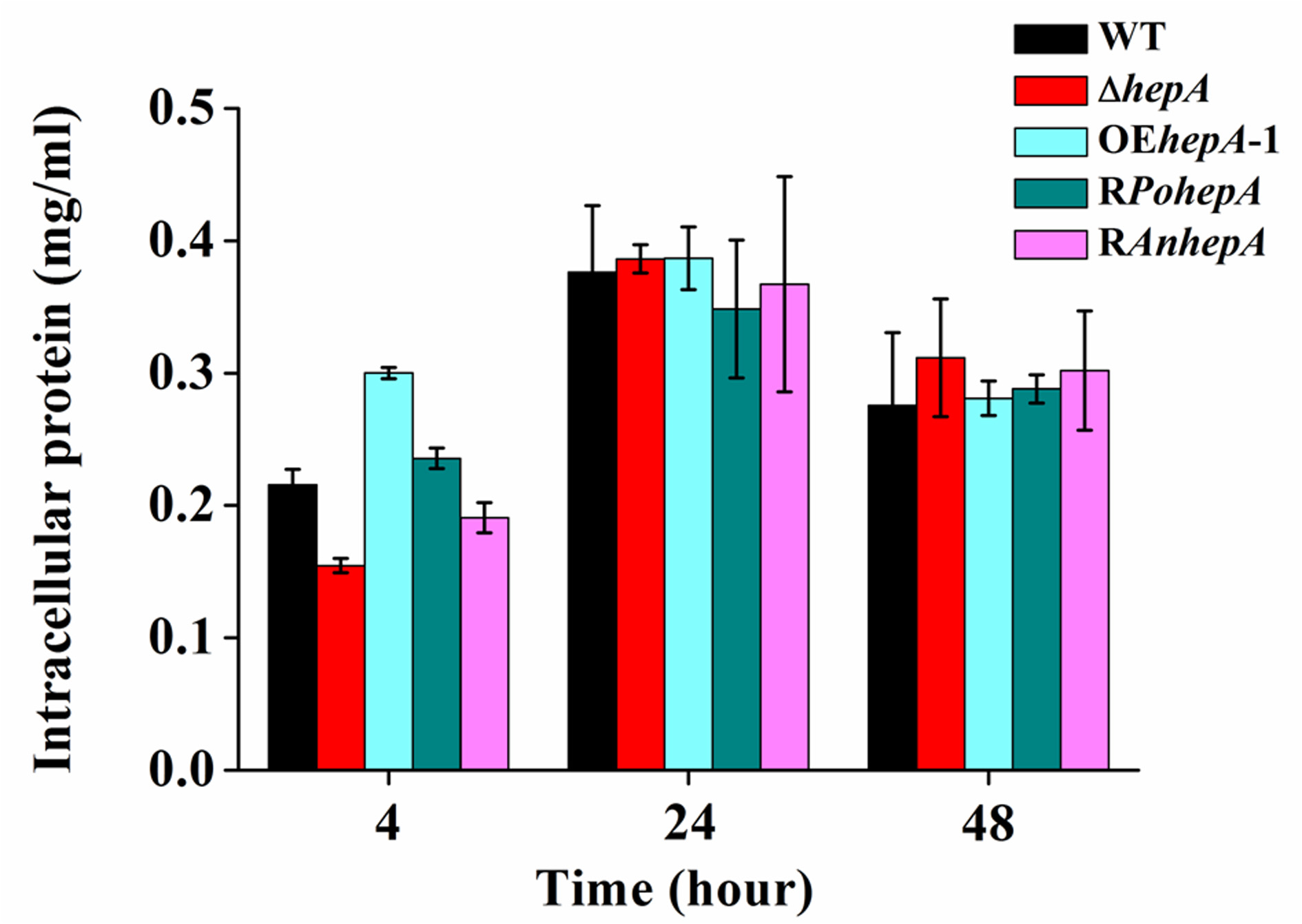

Supplement: Supplementary file 5 — 10.1186/s13068-016-0624-9 Total intracellular protein concentration assay for WT and vavious mutants. [file 13068_2016_624_MOESM5_ESM.jpg]

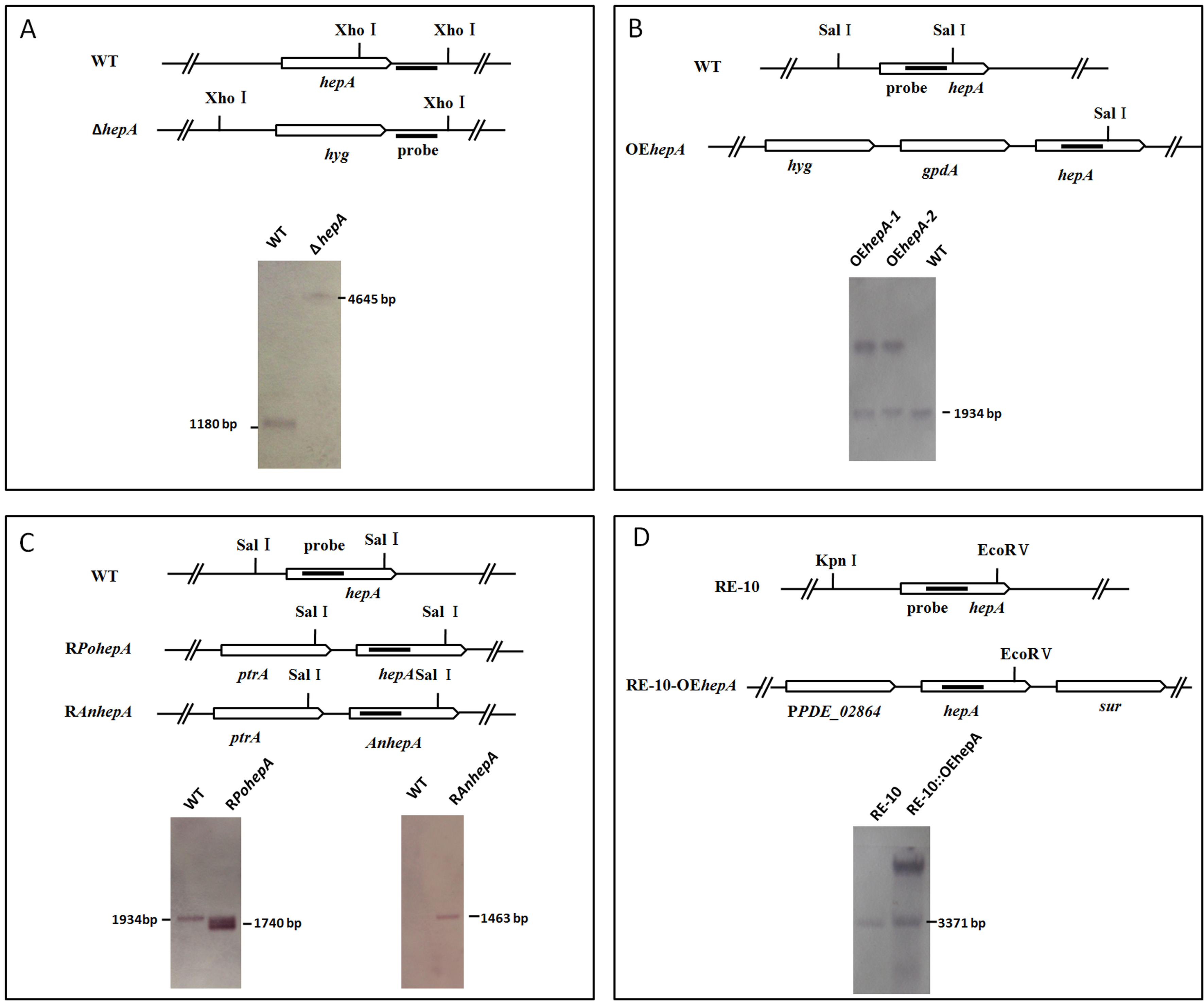

Supplement: Supplementary file 7 — 10.1186/s13068-016-0624-9 Strategies and results of Southern blot for different mutants. (A) hepA deletion strain, ΔhepA. (B) hepA overexpression strain in WT, OEhepA. (C) P. oxalicum hepA recomplement strain, RPohepA, and A. nidulans hepA recomplement strain, RAnhepA. (D) hepA overexpression strain in RE-10, RE-10::OEhepA. [file 13068_2016_624_MOESM7_ESM.jpg]
