# Supplementary material for: Expression and chromatin structures of cellulolytic enzyme gene regulated by heterochromatin protein 1
Source: Biotechnol Biofuels. 2016 Oct 3;9:206. doi: 10.1186/s13068-016-0624-9 (PMC5048463; doi:10.1186/s13068-016-0624-9)
Supplement: Supplementary file 2 — 10.1186/s13068-016-0624-9 List of upregulated genes (≥twofold, FDR < 0.05) in ∆hepA compared with WT with significantly enriched GO terms (GO category: molecular function). [file 13068_2016_624_MOESM2_ESM.docx]

**Table S1** List of upregulated genes (≥ twofold, FDR < 0.05) in Δ*hepA* compared with WT with significantly enriched GO terms (GO category: molecular function)

| **GO-ID** | **Term** | **Gene ID**  **(locus_tag)** | **Description of putative *P. oxalicum* ORF** |
| --- | --- | --- | --- |
| GO:0016597 | Amino acid binding | PDE_02311 | Probable aspartokinase |
|  |  | PDE_02324 | D-3-phosphoglycerate dehydrogenase |
|  |  | PDE_03642 | Ornithine carbamoyltransferase |
|  |  | PDE_03644 | Acetolactate synthase small subunit |
|  |  | PDE_07967 | Aspartate carbamoyltransferase |
| GO:0015171 | Amino acid transmembrane transporter activity | PDE_00281 | Dicarboxylic amino acid permease |
|  |  | PDE_00421 | Choline transport protein |
|  |  | PDE_00716 | Polyamine transporter |
|  |  | PDE_00845 | General amino-acid permease |
|  |  | PDE_01350 | Choline transport protein |
|  |  | PDE_01623 | Choline transport protein |
|  |  | PDE_03500 | Choline transport protein |
|  |  | PDE_04267 | Uncharacterized amino-acid permease |
|  |  | PDE_04432 | Dicarboxylic amino acid permease |
|  |  | PDE_06022 | Uncharacterized amino-acid permease |
|  |  | PDE_09031 | General amino acid permease |
|  |  | PDE_09138 | Choline transport protein |
|  |  | PDE_09143 | Arginine permease |
|  |  | PDE_09323 | GABA-specific permease |
|  |  | PDE_09673 | Proline-specific permease |
|  |  | PDE_09869 | Uncharacterized amino-acid permease |
| GO:0008026 | ATP-dependent helicase activity | PDE_00048 | ATP-dependent RNA helicase dbp6 |
|  |  | PDE_00396 | ATP-dependent RNA helicase rok1 |
|  |  | PDE_00474 | ATP-dependent RNA helicase mss116 |
|  |  | PDE_00546 | ATP-dependent RNA helicase drs1 |
|  |  | PDE_01963 | ATP-dependent RNA helicase eIF4A |
|  |  | PDE_02617 | ATP-dependent rRNA helicase rrp3 |
|  |  | PDE_03690 | ATP-dependent RNA helicase ded1 |
|  |  | PDE_03703 | ATP-dependent RNA helicase mss116 |
|  |  | PDE_03848 | ATP-dependent RNA helicase dbp2 |
|  |  | PDE_04191 | ATP-dependent RNA helicase mrh4 |
|  |  | PDE_05032 | ATP-dependent RNA helicase dbp8 |
|  |  | PDE_05104 | ATP-dependent rRNA helicase spb4 |
|  |  | PDE_06233 | ATP-dependent RNA helicase dbp3 |
|  |  | PDE_06270 | DNA repair protein RAD16 |
|  |  | PDE_07529 | Pre-mRNA-splicing factor ATP-dependent RNA helicase PRP43 |
|  |  | PDE_07599 | ATP-dependent RNA helicase dbp7 |
|  |  | PDE_07986 | ATP-dependent RNA helicase dbp10 |
|  |  | PDE_07992 | ATP-dependent RNA helicase dbp4 |
|  |  | PDE_08321 | ATP-dependent RNA helicase has1 |
|  |  | PDE_08403 | ATP-dependent RNA helicase dbp9 |
|  |  | PDE_08790 | ATP-dependent RNA helicase mak5 |
|  |  | PDE_09906 | ATP-dependent RNA helicase fal1 |
| GO:0016614 | Oxidoreductase activity, acting on CH-OH group of donors | PDE_00027 | Long-chain-alcohol oxidase |
|  |  | PDE_00035 | 2-hydroxyacid dehydrogenase homolog |
|  |  | PDE_00171 | Rhamnolipids biosynthesis 3-oxoacyl-[acyl-carrier-protein] reductase |
|  |  | PDE_00366 | Isocitrate dehydrogenase [NAD] subunit 2 |
|  |  | PDE_00542 | 3-isopropylmalate dehydrogenase A |
|  |  | PDE_00543 | Putative D-arabinono-1,4-lactone oxidase |
|  |  | PDE_00966 | Choline dehydrogenase |
|  |  | PDE_01202 | NADP-dependent alcohol dehydrogenase 6 |
|  |  | PDE_01324 | Cytochrome b2 |
|  |  | PDE_01389 | Mannitol 2-dehydrogenase |
|  |  | PDE_01413 | Glucose oxidase |
|  |  | PDE_01734 | NADP-dependent alcohol dehydrogenase 6 |
|  |  | PDE_02091 | Ketol-acid reductoisomerase |
|  |  | PDE_02172 | UDP-glucose 6-dehydrogenase |
|  |  | PDE_02324 | D-3-phosphoglycerate dehydrogenase 2 |
|  |  | PDE_02350 | NAD/NADP-dependent indole-3-acetaldehyde reductase |
|  |  | PDE_02494 | Isocitrate dehydrogenase |
|  |  | PDE_03317 | Alcohol dehydrogenase |
|  |  | PDE_03406 | 2-dehydropantoate 2-reductase |
|  |  | PDE_04142 | NADP-dependent malic enzyme |
|  |  | PDE_04218 | Uncharacterized oxidoreductase yxbG |
|  |  | PDE_04537 | Glycerol-3-phosphate dehydrogenase |
|  |  | PDE_04628 | Uncharacterized oxidoreductase dltE |
|  |  | PDE_04799 | Homoserine dehydrogenase |
|  |  | PDE_05183 | Probable homoisocitrate dehydrogenase |
|  |  | PDE_05317 | Probable inosine-5&apos;-monophosphate dehydrogenase |
|  |  | PDE_05556 | Probable cinnamyl alcohol dehydrogenase 2 |
|  |  | PDE_05973 | 6-hydroxy-D-nicotine oxidase |
|  |  | PDE_06079 | Zinc-type alcohol dehydrogenase-like protein |
|  |  | PDE_06534 | Sterol-4-alpha-carboxylate 3-dehydrogenase |
|  |  | PDE_06831 | Glycerol-3-phosphate dehydrogenase |
|  |  | PDE_06915 | Uncharacterized oxidoreductase |
|  |  | PDE_07438 | Rhamnolipids biosynthesis 3-oxoacyl-[acyl-carrier-protein] reductase |
|  |  | PDE_07603 | U2 small nuclear ribonucleoprotein |
|  |  | PDE_07606 | 6-hydroxy-D-nicotine oxidase |
|  |  | PDE_07636 | Uncharacterized oxidoreductase |
|  |  | PDE_07692 | D-arabinitol 2-dehydrogenase |
|  |  | PDE_07857 | Alcohol dehydrogenase [acceptor] |
|  |  | PDE_08180 | 6-hydroxy-D-nicotine oxidase |
|  |  | PDE_08693 | Bifunctional protein RIB2 |
|  |  | PDE_09239 | Versicolorin reductase |
|  |  | PDE_09616 | Alcohol dehydrogenase |
|  |  | PDE_09681 | Peroxisomal 2,4-dienoyl-CoA reductase |
|  |  | PDE_09770 | D-arabinitol 2-dehydrogenase |
| GO:0008173 | RNA methyltransferase activity | PDE_01448 | N2,N2-dimethylguanosine tRNA methyltransferase |
|  |  | PDE_06193 | Dimethyladenosine transferase |
|  |  | PDE_07485 | AdoMet-dependent rRNA methyltransferase spb1 |
|  |  | PDE_07537 | Uncharacterized RNA methyltransferase |
|  |  | PDE_08307 | rRNA methyltransferase |
|  |  | PDE_08517 | tRNA (guanine-N(7)-)-methyltransferase |
|  |  | PDE_08617 | tRNA (adenine-N(1)-)-methyltransferase |
|  |  | PDE_08789 | Putative ribosomal RNA methyltransferase C4F |
|  |  | PDE_09566 | tRNA (adenine-N(1)-)-methyltransferase catalytic subunit trm61 |
| GO:0019843 | rRNA binding | PDE_00374 | 60S ribosomal protein L5 |
|  |  | PDE_00734 | 37S ribosomal protein MRP17 |
|  |  | PDE_01806 | 40S ribosomal protein S9 |
|  |  | PDE_01824 | 60S ribosomal protein L37 |
|  |  | PDE_03221 | 54S ribosomal protein L16 |
|  |  | PDE_04146 | U3 small nucleolar ribonucleoprotein protein IMP3 |
|  |  | PDE_06792 | 60S ribosomal protein L9-B |
| GO:0003735 | Structural constituent of ribosome | PDE_00304 | 60S ribosomal protein L17 |
|  |  | PDE_00374 | 60S ribosomal protein L5 |
|  |  | PDE_00406 | 40S ribosomal protein S5 |
|  |  | PDE_00491 | 40S ribosomal protein S22 |
|  |  | PDE_00629 | 60S ribosomal protein L6-B |
|  |  | PDE_00734 | 37S ribosomal protein MRP17 |
|  |  | PDE_00817 | 40S ribosomal protein S28 |
|  |  | PDE_01007 | Probable 60S ribosomal protein L28e |
|  |  | PDE_01019 | 30S ribosomal protein S10 |
|  |  | PDE_01156 | 40S ribosomal protein S9 |
|  |  | PDE_01517 | 60S ribosomal protein L27a |
|  |  | PDE_01695 | 40S ribosomal protein S20 |
|  |  | PDE_01806 | 40S ribosomal protein S9 |
|  |  | PDE_01807 | 60S ribosomal protein L21 |
|  |  | PDE_01817 | 40S ribosomal protein S4 |
|  |  | PDE_01824 | 60S ribosomal protein L37 |
|  |  | PDE_01832 | 40S ribosomal protein S27 |
|  |  | PDE_02248 | 60S ribosomal protein L39 |
|  |  | PDE_02446 | 54S ribosomal protein L12 |
|  |  | PDE_02590 | 60S acidic ribosomal protein P1 |
|  |  | PDE_02599 | 60S ribosomal protein L27-A |
|  |  | PDE_02649 | 37S ribosomal protein MRP4 |
|  |  | PDE_02687 | 60S ribosomal protein L26-B |
|  |  | PDE_02693 | 40S ribosomal protein S26E |
|  |  | PDE_02707 | 60S ribosomal protein L16 |
|  |  | PDE_02754 | 40S ribosomal protein S3 |
|  |  | PDE_02775 | 40S ribosomal protein S12 |
|  |  | PDE_02793 | 40S ribosomal protein S19 |
|  |  | PDE_02848 | Ubiquitin-60S ribosomal protein L40 |
|  |  | PDE_02864 | 40S ribosomal protein S8 |
|  |  | PDE_02907 | 60S ribosomal protein L15 |
|  |  | PDE_02916 | 60S ribosomal protein L20 |
|  |  | PDE_03013 | 60S ribosomal protein L29 |
|  |  | PDE_03085 | 54S ribosomal protein L4 |
|  |  | PDE_03221 | 54S ribosomal protein L16 |
|  |  | PDE_03397 | 60S ribosomal protein L24 |
|  |  | PDE_03512 | 60S ribosomal protein L14-A |
|  |  | PDE_03668 | 60S ribosomal protein L36 |
|  |  | PDE_03684 | 40S ribosomal protein L34 |
|  |  | PDE_03779 | 60S acidic ribosomal protein P2 |
|  |  | PDE_03780 | 40S ribosomal protein S15 |
|  |  | PDE_03793 | 40S ribosomal protein S17 |
|  |  | PDE_03807 | 40S ribosomal protein S14 |
|  |  | PDE_04060 | 60S ribosomal protein L18-B |
|  |  | PDE_04132 | 60S ribosomal protein L44 |
|  |  | PDE_04197 | 54S ribosomal protein L38 |
|  |  | PDE_04327 | 60S ribosomal protein L10-A |
|  |  | PDE_04328 | 60S ribosomal protein L30 |
|  |  | PDE_04761 | 39S ribosomal protein L49 |
|  |  | PDE_04825 | 60S ribosomal protein L22 |
|  |  | PDE_04897 | Ubiquitin-40S ribosomal protein S27a |
|  |  | PDE_04939 | 40S ribosomal protein MRP2 |
|  |  | PDE_05026 | 54S ribosomal protein L22 |
|  |  | PDE_05144 | 40S ribosomal protein S7 |
|  |  | PDE_05179 | 37S ribosomal protein S8 |
|  |  | PDE_05203 | 40S ribosomal protein S11 |
|  |  | PDE_05253 | 60S ribosomal protein L34 |
|  |  | PDE_05292 | 60S ribosomal protein L23 |
|  |  | PDE_05315 | 40S ribosomal protein S21 |
|  |  | PDE_05375 | Ribosome biogenesis protein RLP24 |
|  |  | PDE_05635 | 60S ribosomal protein L38 |
|  |  | PDE_05649 | 60S ribosomal protein L11 |
|  |  | PDE_05678 | 60S ribosomal protein L7 |
|  |  | PDE_05983 | 40S ribosomal protein S2 |
|  |  | PDE_06186 | 37S ribosomal protein RSM18 |
|  |  | PDE_06755 | 40S ribosomal protein S23 |
|  |  | PDE_06758 | 60S ribosomal protein L8 |
|  |  | PDE_06825 | Probable 60S ribosomal protein L32 |
|  |  | PDE_06792 | 60S ribosomal protein L9-B |
|  |  | PDE_07189 | 60S ribosomal protein L35 |
|  |  | PDE_07395 | 60S ribosomal protein yml6 |
|  |  | PDE_07519 | 54S ribosomal protein L23 |
|  |  | PDE_07536 | Probable 40S ribosomal protein S5 |
|  |  | PDE_07774 | 60S ribosomal protein L32 |
|  |  | PDE_07853 | 60S ribosomal protein L43 |
|  |  | PDE_08100 | 40S ribosomal protein S18 |
|  |  | PDE_08142 | 60S ribosomal protein L31 |
|  |  | PDE_08177 | 37S ribosomal protein S17 |
|  |  | PDE_08205 | 40S ribosomal protein S29 |
|  |  | PDE_09073 | 54S ribosomal protein L9 |
|  |  | PDE_09221 | Putative ribosome biogenesis protein C8F11.04 |
|  |  | PDE_09361 | 60S ribosomal protein L2 |
|  |  | PDE_09511 | 50S ribosomal protein L1 |
|  |  | PDE_09556 | 60S ribosomal protein L2 |
|  |  | PDE_09728 | 60S ribosomal protein L30-1 |
|  |  | PDE_09844 | 54S ribosomal protein IMG1 |
|  |  |  |  |
