# Supplementary material for: Expression and chromatin structures of cellulolytic enzyme gene regulated by heterochromatin protein 1
Source: Biotechnol Biofuels. 2016 Oct 3;9:206. doi: 10.1186/s13068-016-0624-9 (PMC5048463; doi:10.1186/s13068-016-0624-9)
Supplement: Supplementary file 6 — 10.1186/s13068-016-0624-9 Primers used in this study. [file 13068_2016_624_MOESM6_ESM.docx]

**Table S2** Primers used in this study

| **Primers** | **Sequences** | |
| --- | --- | --- |
| For *hepA* deletion | | |
| DhepA-UF | | CTGGAAGCTTGTTCTGAAGAAG |
| DhepA-UR | | GAATCAGGGGATAACGCAGGAAAGAGTGAAGTTAAGAGGCCATACAG |
| DhepA-DF | | GTTATTCGGCCTACCTGGCTGTGCTGACTGGTGTATTTGTTCATCATC |
| DhepA-DR | | CTCGAGCAAGATGCAGATACCATTC |
| hph-F | | TCTTTCCTGCGTTATCCCCTGATTC |
| hph-R | | CACAGCCAGGTAGGCCGAATAAC |
| DhepA-NF | | GAACCAGAAATGTGATCGTACATC |
| DhepA-NR | | AACATACTTACTCGGTCAGGTC |
| For *P. oxalicum* and *A. nidulans laeA* complement | | |
| ptra-F | | GGGCAATTGATTACGGGATCCCATT |
| ptra-R | | ATGGGGTGACGATGAGCCGCTCTTG |
| RhepA-F | | GCAAGAGCGGCTCATCGTCACCCCATGTCAGCATTGATCTCAGTATG |
| RhepA-R | | TTAGAGAGTGGGAATCCACTTC |
| RhepA-NF | | GATGTCAGGATGATTTCACTTGTTTC |
| RhepA-NR | | GTTACAGGCTCGTTGGGAGTAC |
| ANhepA-F | | CAAGAGCGGCTCATCGTCACCCCATGATAGTAGCACCATCGTTCGTAAC |
| ANhepA-R | | GAATCTAACACAAGTATTGACAG |
| ANhepA-NF | | ATGTAAAAGCTAGGAGATCGTC |
| ANhepA-NR | | TTTCTGGAGCTACCATTCCTC |
| For *hepA* overexpression in WT or in RE-10 | | |
| gpdA-U | | GTTATTCGGCCTACCTGGCTGTGTGAACTGGACGGGAAGGCACT |
| gpdA-D | | GAAATATCTTCAATAGGGGGAGGCATTTTTGCGATTGTTTGAAGTGTTCTG |
| OEhepA-U | | ATGCCTCCCCCTATTGAAGATATTTC |
| OEhepA-D | | TTAGAGAGTGGGAATCCACTTC |
| OEhepA-NU | | CGGATAACAATTTCACACAGGAAACAG |
| OEhepA-ND | | GTTACAGGCTCGTTGGGAGTAC |
| DF | | CGCCGATTGCCTTGATACGT |
| DR | | TTTGCGATATGATGGAGGGGTC |
| REhepA-F | | GACCCCTCCATCATATCGCAAAATGCCTCGTGAGTGACTGCGTC |
| REhepA-R | | TGGCGTTGGCACGTCGACGAGGATTTGTTCCAATGAAG |
| Sur-F | | GTCGACGTGCCAACGCCA |
| Sur-R | | GTCGACGTGAGAGCATGCAATTCC |
| RE-NF | | CTGGGTTTCTTGCCTGAGTT |
| RE-NR | | GTCGACGTGAGAGCATGCAATTCC |
| For Southern blot | | |
| DhepA-SU | | TCATGAGCAGGATCTACGACAG |
| DhepA-SD | | GACTCAAGCAACTACGAGTTG |
| OEhepA-SU | | GCAAACGACGATGTGATCGAG |
| OEhepA-SD | | TCCAGCTTTGGCGACTGGTTTC |
| PohepA-SU | | CTCTTAACTTCACGCTGTTCTC |
| PohepA-SD | | GATTTTCTTGTAATAAGCGTC |
| For real time qPCR of genes | | |
| CBH-QF | | CCACCACCACTACCAGCAAGG |
| CBH-QR | | GTAGCCAACACCACCGCACT |
| BG-QF | | CACCAACACCGGCTCAGTTA |
| BG-QR | | GGACATCCCAGTTGGACAGAT |
| EG-QF | | ACCGCTGCTCAGACCACGAC |
| EG-QR | | TGGGTCCCGAGTAGCCAACG |
| Act-QF | | GTTCCATTCTCGCCTCCCTCT |
| Act-QR | | AGAAGCACTTGCGGTGAACGA |
| creA-QF | | TGGGTACGAGTGAACTCCATCTT |
| creA-QR | | TGTGACCTTGACCAGGACTGTAA |
| clrB-QF | | AGCACAAGTCGAGATGGGATT |
| clrB-QR | | CGCTTGCTGGCTTCGTAAAT |
| bgl2-QF | | GGCTGATGCGTACACGTTTGA |
| bgl2-QR | | CGACATAAGTCACGCCGAAGC |
| For CHART PCR analysis of the core promoter regions | | |
| CBH1-F2 | | TGCACAGTGGTGCGGACC |
| CBH1-R2 | | AGGAATGGATGATGAACACCA |
| CBH1-F3 | | CACTGCAAAAGGACTGGAC |
| CBH1-R3 | | AGCCCCAGGGGGTGAAGTTC |
| CBH1-F4 | | ATCAAGGCACATTGTCATTTG |
| CBH1-R4 | | CTTTCATCTCCCCCAGAGTC |
| EG1-F3 | | GCTTACACTCATCATGCGCA |
| EG1-R3 | | GAGTTTGTGGAGTTGAAGTG |
| EG1-F2 | | GACGCAGTCACGCTAGTGAG |
| EG1-R2 | | GAGCGTGGGTCCATGTATCAG |
| EG1-F1 | | CATGTCTGCAGAATTCTCC |
| EG1-R1 | | ATGCAGACTCGGTGGATGG |
